# Bacterial volatile organic compounds (VOCs) promote growth and induce metabolic changes in rice

**DOI:** 10.3389/fpls.2022.1056082

**Published:** 2023-02-09

**Authors:** Octávio Augusto Costa Almeida, Natália Oliveira de Araujo, Aline Tieppo Nogueira Mulato, Gabriela Felix Persinoti, Maurício Luís Sforça, Maria Juliana Calderan-Rodrigues, Juliana Velasco de Castro Oliveira

**Affiliations:** ^1^ Brazilian Biorenewables National Laboratory (LNBR), Brazilian Center for Research in Energy and Materials (CNPEM), Campinas, Brazil; ^2^ Graduate Program in Genetics and Molecular Biology, Institute of Biology, University of Campinas (UNICAMP), Campinas, Brazil; ^3^ Brazilian Biosciences National Laboratory, Brazilian Center for Research in Energy and Materials (CNPEM), Campinas, Brazil; ^4^ Group of Metabolic Regulation of Plant Growth, Max Planck Institute of Molecular Plant Physiology, Postdam, Germany

**Keywords:** microbial volatile organic compounds, *Oryza sativa*, plant growth promotion, bioactive compounds, metabolomics

## Abstract

Plant growth-promoting bacteria (PGPB) represent an eco-friendly alternative to reduce the use of chemical products while increasing the productivity of economically important crops. The emission of small gaseous signaling molecules from PGPB named volatile organic compounds (VOCs) has emerged as a promising biotechnological tool to promote biomass accumulation in model plants (especially *Arabidopsis thaliana*) and a few crops, such as tomato, lettuce, and cucumber. Rice (*Oryza sativa*) is the most essential food crop for more than half of the world’s population. However, the use of VOCs to improve this crop performance has not yet been investigated. Here, we evaluated the composition and effects of bacterial VOCs on the growth and metabolism of rice. First, we selected bacterial isolates (IAT P4F9 and E.1b) that increased rice dry shoot biomass by up to 83% in co-cultivation assays performed with different durations of time (7 and 12 days). Metabolic profiles of the plants co-cultivated with these isolates and controls (without bacteria and non-promoter bacteria—1003-S-C1) were investigated *via* ^1^H nuclear magnetic resonance. The analysis identified metabolites (e.g., amino acids, sugars, and others) with differential abundance between treatments that might play a role in metabolic pathways, such as protein synthesis, signaling, photosynthesis, energy metabolism, and nitrogen assimilation, involved in rice growth promotion. Interestingly, VOCs from IAT P4F9 displayed a more consistent promotion activity and were also able to increase rice dry shoot biomass *in vivo*. Molecular identification by sequencing the 16S rRNA gene of the isolates IAT P4F9 and E.1b showed a higher identity with *Serratia* and *Achromobacter* species, respectively. Lastly, volatilomes of these and two other non-promoter bacteria (1003-S-C1 and *Escherichia coli* DH5α) were evaluated through headspace solid-phase microextraction coupled with gas chromatography–mass spectrometry. Compounds belonging to different chemical classes, such as benzenoids, ketones, alcohols, sulfide, alkanes, and pyrazines, were identified. One of these VOCs, nonan-2-one, was validated *in vitro* as a bioactive compound capable of promoting rice growth. Although further analyses are necessary to properly elucidate the molecular mechanisms, our results suggest that these two bacterial isolates are potential candidates as sources for bioproducts, contributing to a more sustainable agriculture.

## 1 Introduction

Microbial volatile organic compounds (VOCs), produced by organisms such as bacteria and fungi, are small signaling molecules (<C15) having low molecular masses (<300 Da), high vapor pressure, and low boiling point. Usually, these molecules are in the gas phase (at 25°C temperature/1 atm pressure), as their properties facilitate evaporation. VOCs are normally lipophilic compounds and can readily diffuse through water and gas-filled pores in soil and rhizosphere environments (Effmert et al., 2012; Schmidt et al., 2016; Schulz-Bohm et al., 2017). Therefore, differently from soluble metabolites, which are often involved in short-distance interactions, VOCs can reach and act even at long distances (Tyc et al., 2017; Westhoff et al., 2017; Schulz-Bohm et al., 2018).

Approximately 2,000 VOCs emitted from almost 1,000 bacterial and fungal species were already identified (Lemfack et al., 2018). However, the number of microorganisms investigated so far is still small since 10^12^ microbial species are expected to exist on Earth (Locey and Lennon, 2016). Bacterial VOCs belong to different chemical classes including alcohols, ketones, benzenoids, terpenoids, sulfur-containing compounds, alkenes, and others (Schulz and Dickschat, 2007; Peñuelas et al., 2014; Schmidt et al., 2016). The term “volatilome” describes all volatile compounds produced by an organism (Tilocca et al., 2020). Their composition depends on many factors, such as the nutrient medium, pH, aeration, and stage of culture growth. Moreover, some compounds are common in the volatilomes of a whole group of bacteria, but others are specific to particular strains (Schmidt et al., 2015; Hernández-Calderón et al., 2018; Guo et al., 2019).

VOCs are by-products of microbial metabolism, but still play important roles in intra- and inter-kingdom interactions. Bacterial VOCs can have antagonistic effects against other microorganisms (Raza et al., 2016a; Raza et al., 2016b; Freitas et al., 2022), promote their growth (Ryu et al., 2003; Song et al., 2019), and modulate virulence and resistance to antibiotics, biofilm formation, and motility (Effmert et al., 2012; Schulz-Bohm et al., 2017). These compounds can also benefit plants by serving as a direct nutrient source, modulating phytohormone pathways (such as auxin and cytokinin), increasing nutrient absorption, regulating enzyme activities, or even inducing systemic resistance against biotic and abiotic stressors (Bitas et al., 2013; Kanchiswamy et al., 2015; Fincheira and Quiroz, 2018). The effects of bacterial VOCs were mainly investigated in the model *Arabidopsis thaliana*, revealing an increase in total leaf surface area (Ryu et al., 2003), rosette leaf number, flowering time, biomass weight, seed set number (Xie et al., 2009), and root length (Maheshwari et al., 2021). For instance, Zhang et al. (2007) presented that the mechanism of action of VOCs from *Bacillus amyloliquefaciens* GB03 to promote the growth of *A. thaliana* involved the regulation of auxin homeostasis and cell wall expansion. Despite those beneficial actions of VOCs, some growth inhibition caused by volatiles from *Serratia* species has already been reported in *A. thaliana* (Plyuta et al., 2021). This might justify the commercial potentiality of identifying the effects of VOCs emitted by individual bacteria on plant growth promotion.

Few investigations revealed the VOCs’ effectiveness in non-model species. Volatiles from *Pseudomonas pseudoalcaligenes* incremented biomass, germination, and drought tolerance in maize (*Zea mays* L.) by inducing changes in the defense system (Yasmin et al., 2021). Heenan-Daly et al. (2021) showed that VOCs from *Bacillus* and *Serratia* isolates controlled the expression of genes involved in photosynthetic activity, defense, and stress response in potato (*Solanum tuberosum*). Moreover, the compounds benzaldehyde, 1,2-benzisothiazol-3(2 H)-one, and 1,3-butadiene, emitted by two *Bacillus* species, improved tobacco resistance against wilt disease using a dual approach; reducing *Ralstonia solanacearum* growth while increasing the expression of plant genes involved in the salicylic acid pathway (Tahir et al., 2017a). Although several studies reinforce that VOCs can boost plant growth and health, their mechanisms of action are still poorly understood.

Rice (*Oryza sativa*) is a model monocotyledonous plant and has several specificities in relation to *A. thaliana* (a dicotyledonous) (Izawa and Shimamoto, 1996), such as in the shape of the leaves (Nelissen et al., 2016), branching of root (Pagès, 2016), and seed development (Sreenivasulu and Wobus, 2013). Furthermore, rice represents the main food crop for more than 50% of the world’s population, besides playing an important role in animal feed (Parida et al., 2022). The world rice production in 2021/2022 is estimated at 514.07 million tons (United States Department of Agriculture, 2022). Although productivity per hectare has more than doubled since the 1960s, a further doubling will be necessary to feed the world’s increasing population by 2050. However, rice production during the past decade has almost reached stagnation (Parida et al., 2022). VOC-producing bacteria are thus a promising eco-friendly alternative to increase its productivity (Bitas et al., 2013; Kanchiswamy et al., 2015; Fincheira and Quiroz, 2018; Brilli et al., 2019; Dias et al., 2021). To the best of our knowledge, there is no report about the growth promotion activity of bacterial VOCs on rice.

In this study, we have investigated the metabolome of rice treated with bacterial VOCs and identified two bacteria species causing several metabolic alterations (such as in the energy metabolism) and inducing rice growth. Furthermore, the compound nonan-2-one was detected in the volatilome and was shown to increase rice shoot biomass *in vitro*. VOCs produced by the strain IAT P4F9 could also promote biomass accumulation *in vivo*. This is the first report about the effects of bacterial VOCs on this important crop. Our findings are of particular significance for the agricultural application of VOCs to promote plant growth.

## 2 Materials and methods

### 2.1 Bacterial isolates and plant material

The bacterial strains from sugarcane and energy cane fields (rhizospheric soil and root), as well as from composting of filter cake, were previously isolated in our laboratory (**Supplementary Table 1**). We selected 14 isolates from different genera (formerly identified by sequencing of the 16S rRNA V3–V5 region), source, and location (**Supplementary Table 1**). These bacteria were stored in LB broth supplemented with cryoprotectant solution (25 g L^−1^ gelatin, 50 g L^−1^ lactose, 10 g L^−1^ peptone, and 250 g L^−1^ glycerol) at −80°C. Seeds of rice variety IRGA 424 RI (kindly donated by Corteva Agriscience, Mogi Mirim, Brazil), recommended to be cultivated in irrigated or dry land systems in the southern region of Brazil, were surface-sterilized by soaking in 1% sodium NaClO solution containing 50 µl of Tween-20 emulsifier for 30 min with manual stirring. Seeds were then rinsed eight times with sterile Milli-Q water.

### 2.2 Screening of rice growth-promoting activity

The sterilized rice seeds were placed on Petri dishes (150 × 15 mm) containing half-strength Murashige and Skoog salt (½ MS) medium (Murashige and Skoog, 1962) and 1% agar (pH 5.7) in a plant growth chamber (Fitotron^®^ HGC Weiss Technik) under a 12-h photoperiod (21°C, irradiance of 150 μmol m^−2^ s^−1^/19°C, dark) and a relative humidity of 75%. One day before the co-cultivation experiments, bacterial isolates were grown on LB liquid media for 16 h. The culture was diluted with water to yield 10^8^ CFU ml^−1^ based on optical density. The co-cultivation system consisted of a small Petri dish (49 × 12 mm) containing LB solid media inside a larger Petri dish (150 × 15 mm) filled with ½ MS solid medium (**Supplementary Figure 1**). Two germinated rice seedlings of 4-day-old with the prophyll emergence from the coleoptile (growth stage S3) (Counce et al., 2000) were transferred to the larger Petri dish and 20 µl of the diluted bacterial inoculum were spread onto the small dish. Rice seedlings cultivated on the same system without bacterial inoculum were used as the control, and co-culture plates inoculated with *Escherichia coli* DH5α were used as the negative control since it does not promote plant growth in several reports (Ryu et al., 2003; Ryu et al., 2005; Kanchiswamy et al., 2015; Tahir et al., 2017b; Tahir et al., 2017c; Rath et al., 2018). Plates were sealed with polyvinyl chloride (PVC) film, positioned to form a ~70° angle to support aerial root growth, and placed in the growth chamber for 7 days.

After this period, the dry weight of the roots and shoots was measured on an analytical balance. Morphological parameters of root architecture (total length, primary and secondary root lengths, surface area, and volume) were acquired by imaging using a scanner STD4800 (Regent Instruments Inc, Quebec, Canada), at 4,800 dpi, and the WinRHIZO Regular 2019a software. The experiment was performed in a complete randomized block design with four replicates (in total eight seedlings). Statistical differences for each evaluated parameter were calculated and compared by analysis of variance (ANOVA) and Tukey *post hoc* test (*p* < 0.05) using AgroEstat v.1.1.0.712 software (Barbosa and Maldonado, 2010). Outlier (one seedling) was removed from each treatment.

New co-cultivation assays were performed to evaluate the growth promotion effects in a longer period. For that, the bacterial isolates that promoted the highest increase in rice growth after 7 days of co-cultivation [IAT P4F9 and E.1b(Xilano01_11), hereafter referred to as the short name “E.1b”] were chosen. The experiments were conducted following the same methodology, but the rice dry weight was evaluated after 12 days of co-cultivation.

### 2.3 Metabolome analysis

For metabolome analysis, plants were also co-cultivated with 1003-S-C1 (a bacterium that did not promote rice growth—negative control). The experiments were conducted following the same above-mentioned procedures and controls. The dry weight of shoots and roots from eight plants per treatment was evaluated after 12 days as previously described. Shoots dedicated to metabolomic analysis were harvested (15 per replicate), ground in liquid nitrogen with pestle and mortar, and stored at −80°C until metabolite extraction. The experiment was performed with four replicates.

Metabolites were extracted from 100 mg of frozen tissue with 600 μl of methanol (Sigma-Aldrich) and chloroform (Thermo Fisher Scientific) solution [2:1 (v/v) ratio]. After vortexing for 10 s, samples were sonicated for 5 min in a Branson Bransonic^®^ (Emerson) ultrasonic bath, at room temperature. Samples were then kept on ice for 15 min and 300 μl of chloroform plus 300 μl of ice-cold Milli-Q water were added. Samples were homogenized again by vortexing for 10 s and centrifuged at 14,000 rpm for 20 min at 4°C (Eppendorf^®^ 5430R). Afterward, 300 μl of the upper fraction of the three-phase solution was transferred to a new microtube and dried in a refrigerated concentrator (Refrigerated CentriVap Centrifugal Concentrator, Labconco) at 4°C for 48 h to allow complete methanol removal.

The remaining pellets were resuspended in 600 μl of D_2_O-containing phosphate buffer (0.1 M, pH 7.4) and 0.5 mM of trimethylsilylpropionate (TMSPd_4_). Samples were transferred to a 5-mm NMR tube for immediate acquisition using an Agilent DD2 500-MHz spectrometer (Agilent Technologies Inc., Santa Clara, CA, USA) equipped with a triple-resonance probe at 25°C. ^1^H-NMR spectra acquisition was performed with 256 scans collected with 32 K data points over a spectral width of 8,000 Hz. 2D NMR ^1^H-^1^H-TOCSY spectra were acquired using a spectral width of 8,000 Hz and 128 increments with 56 transients of 2k complex points for each free induction decay. 2D NMR ^1^H-^13^C-HSQC spectra were recorded with a spectral width of 8,000 Hz × 25,133 Hz and 128 increments with 60 transients of 2k complex points. In both 2D NMR spectra and ^1^H-NMR spectra, a 1.5-s relaxation delay was incorporated between scans with a continual water pre-saturation radiofrequency (RF) field to eliminate residual water signal.

The metabolites were processed and quantified using NMR Suite software version 8.1 (Chenomx Inc™, Edmonton, AB, Canada). The *Processor* module of this software was used to adjust the spectral phase and for baseline corrections. A 0.5-Hz line-broadening function was used to reduce signal noise and facilitate the fitting of the metabolite signals in spectral peaks. The water signal was suppressed, and the spectra were calibrated using the reference signal of the TMSP-d_4_ as 0.5 mM. The spectra were individually transferred to the *Profiling* module of this software to determine the metabolomic profile of each sample. Metabolites were identified and their concentrations were measured and exported to Excel^®^ (Microsoft Office*™ 365*) and normalized by the fresh weight of the samples. When necessary, the 2D ^1^H-^1^H-TOCSY/^1^H-^13^C-HSQC spectra were used to confirm the identity of some metabolites. Afterward, the metabolome data were analyzed using MetaboAnalyst 5.0 (Pang et al., 2021) following these procedures: data were normalized with Log_10_ transformation, statistical analysis was performed by calculating significance with ANOVA and pairwise comparing mean differences with *t*-test (*p* < 0.05), corrected with false discovery rates (FDRs), principal component analysis (PCA), and hierarchical clustering (HCA) dendrogram based on Pearson distance measure and Ward clustering algorithm, and generation of a heatmap using the normalized data without standardization and with Pearson distance measure and Ward clustering algorithm. Outliers detected by the *RandomForest* function were removed from analyses. The module “Pathway analysis” at MetaboAnalyst 5.0 was used to identify which metabolic pathways have been affected. Data were normalized with Log_10_ transformation, and no scaling was used. The parameter settings were as follows: visualization method as scatter plot (testing significant features), global test for the enrichment method, relative-betweenness centrality for the topology analysis, and the pathway library of *Oryza sativa* subsp. *japonica* (Japanese rice) (KEGG) as the reference metabolome. MetaboAnalyst 5.0 scores of a metabolic pathway may have been impacted from 0 to 1.0; thus, we considered those pathways with significance (*p* < 0.05) and an impact value higher than 0.1.

### 2.4 *In vivo* rice and bacteria co-cultivation

Bacteria and rice co-cultivation in a semi-open system were adapted from Park et al. (2015) (**Supplementary Figure 2**). Five-day-old rice seedlings (decontamination and germination as described in *Section 2.1*) were individually placed in a sterile plastic cup (pre-immersed in a 1% NaClO solution) filled with 70 ml of fine sterile vermiculite. About 10 holes were made at the bottom of the cups (protected with a piece of drainage blanket) to allow the bacterial VOCs to permeate through the substrate. The cups were then inserted into 115-ml glass bottles containing 5 ml of LB solid medium inoculated with 20 µl of 10^8^ CFU of rice growth promoter bacterial isolates IAT P4F9, E.1b, or 1003-S-C1 (negative control). Additional controls were co-cultivation systems with LB medium without bacterial inoculum and inoculated with the growth-promoting bacterium *B. amyloliquefaciens* GB03 (positive control) (Ryu et al., 2004; Xie et al., 2009; Kwon et al., 2010; Delaplace et al., 2015). Rice was cultivated in a growth chamber under a 12-h photoperiod (as described in *Section 2.2*) for 15 days. As we used the rice cultivation system in dry soil, that period was established considering the rice growth limit stages (V3/V4) that precede the recommendation to flooding the field in the Brazilian context (SOSBAI, 2018). Bacterial inoculums were changed every 4 days of co-cultivation to maintain constant levels of VOCs within the system. During the experiment, irrigation was performed daily with 5 ml of sterile distilled water, and plants received 5 ml of Hoagland solution (Hoagland and Arnon, 1950) every 4 days. The dry shoot and root biomass of four plants per treatment were evaluated as described in *Section 2.2*.

### 2.5 Bacterial isolate identification

Improved identification of the best promoter bacteria (ITA P4F9 and E.1b) and the 1003-S-C1 (non-growth-promoting isolate) were performed according to Freitas et al. (2022). Briefly, the bacterial 16S rRNA gene was amplified using the combination of the primers 20F (5´ GAGTTTGAT CCTGGCTCAG 3´) and 1500R (5´ GTTACCTTGTTACG ACTT 3´) and sequenced using 20F, 1500R, 520F (5´ CAGCAGCCG CGGTAATAC 3´), 520R (5´ GTATTACCGCGGCTGCTG 3´), 920F (5´ AAACTCAAATGAATTGACGG 3´), and 920R (5´ CCGTCAATTCATTTGAGTTT 3´) (Yukphan et al., 2004). The obtained contigs were compared with the EzTaxon database (Yoon et al., 2017). All 16S rRNA sequences were deposited in NCBI under accession numbers OP186019 [E.1b(Xilano01_11)], OP186020 (IAT P4F9), and OP186021 (1003-S-C1). In addition, phylogenetic analysis was performed. The 16S rRNA gene sequences of IAT P4F9, 1003-S-C1, and E.1b bacterial isolates were submitted to the online tool 16S-based ID available at the EzBioCloud server (Yoon et al., 2017) to identify 16S rRNA sequences of closely related taxa. These sequences were obtained from either the GenBank database (http://blast.ncbi.nlm.nih.gov/Blast.cgi) or the EzBioCloud server. In addition, the *rpoB* gene of IAT P4F9 and 1003-S-C1 was amplified and sequenced using the primers CM7 (5’ AACCAGTTCCGCGTTGGCCTGG 3’) and CM31 (5’ CCTGAACAACACGCTCGGA 3’) (Mollet et al., 1997), respectively. The *rpoB* sequences were deposited in NCBI under accession numbers OP893533 (IAT P4F9) and OP893532 (1003-S-C1). Multiple sequence alignments of each marker were performed using Mafft v7.310 with parameters –maxiterate 1000 and –localpair (Katoh and Standley, 2013). Maximum likelihood phylogenetic trees were estimated using IQTree software v 2.0.3 using a supermatrix combining the 16S rRNA gene and *rpoB* alignments with 1000 Fastboostrap option. The evolutionary model TIM3+F+I+G4 was chosen according to BIC criteria for the IAT P4F9 and 1003-S-C1 strains. For the E.1b strain the 16S gene sequences were used and the model TN+F+I+G4 was chosen also based on BIC criteria. Phylogenetic trees were analyzed and visualized using the Itol web tool (Letunic and Bork, 2021).

### 2.6 Volatilome analysis

Identification of volatiles emitted from the bacteria identified in *Section 2.5*, as well as from the *E. coli* DH5α strain, was performed following Freitas et al. (2022). Succinctly, volatiles were extracted from bacterial cultures (five replicates per strain) in LB solid medium by the solid phase microextraction (SPME) method using a DVB/CAR/PDMS fiber (Supelco^®^, Sigma Aldrich). The fiber was conditioned at 250°C for 5 min in the hot injector and exposed in culture vials at 50°C for 30 min. After extraction, the SPME fiber was directly inserted into the front inlet of a gas chromatograph (Agilent 7890A, Agilent Technologies) connected to a mass spectrometer (Pegasus^®^ HT TOFMS, LECO Corporation) and desorbed at 250°C for 5 min. The chromatography column was the DB5 (30 m × 0.25 mm × 0.25 µm) (Agilent Technologies) and the temperature of the oven was 30°C for 2 min, 30–300°C at a rate of 20°C min^−1^, 300°C for 2 min, followed by a ramp of 20°C min^−1^ until 330°C for 30 s. NIST v.17 (NIST/EPA/NIH Mass Spectral Library) and an in-house library were used to identify volatiles. The volatilomes were analyzed using MetaboAnalyst v5.0 (Pang et al., 2021) by following the same procedures described in *Section 2.3*, except for the statistical analysis, which was performed with ANOVA and comparing mean differences with Tukey’s test (*p* < 0.05).

### 2.7 *In vitro* validation of VOCs’ effects on rice

Some of the identified VOCs able to promote rice growth were validated using a co-cultivation system prepared as described in *Sections 2.1* and *2.2*. Instead of the bacterial inoculum, solutions with commercial synthetic compounds were applied onto a sterile qualitative filter paper placed in the smaller compartment. Six VOCs [(methyldisulfanyl)methane, (methyltrisulfanyl)methane, heptan-2-one, nonan-2-ol, nonan-2-one, and undecane-2-one] were selected based on the abundance, frequency, and chemical classes of compounds from the best promoter bacteria (ITA P4F9 and E.1b) volatilome profiles on. Compounds were tested at doses of 1.0, 0.1, and 0.01 mg diluted in ethanol, at a final volume of 20 μl. Two mixed solutions were also tested containing three VOCs each: the first one with (methyltrisulfanyl)methane (0.1 mg), nonan-2-one (0.1 mg), and undecane-2-one (0.1 mg), and the second one with (methyldisulfanyl)methane (1.0 mg), heptan-2-one (0.1 mg), and nonan-2-ol (0.1 mg). As a negative control, 20 µl of pure ethanol were used. After 12 days of co-cultivation in the growth chamber under the same conditions described in *Section 2.2*, the shoot and root dry biomass of eight plants per treatment were measured by an analytical balance and evaluated as described in *Section 2.2*.

## 3 Results

### 3.1 Bacterial VOCs increased rice dry shoot biomass

**Figure 1:**
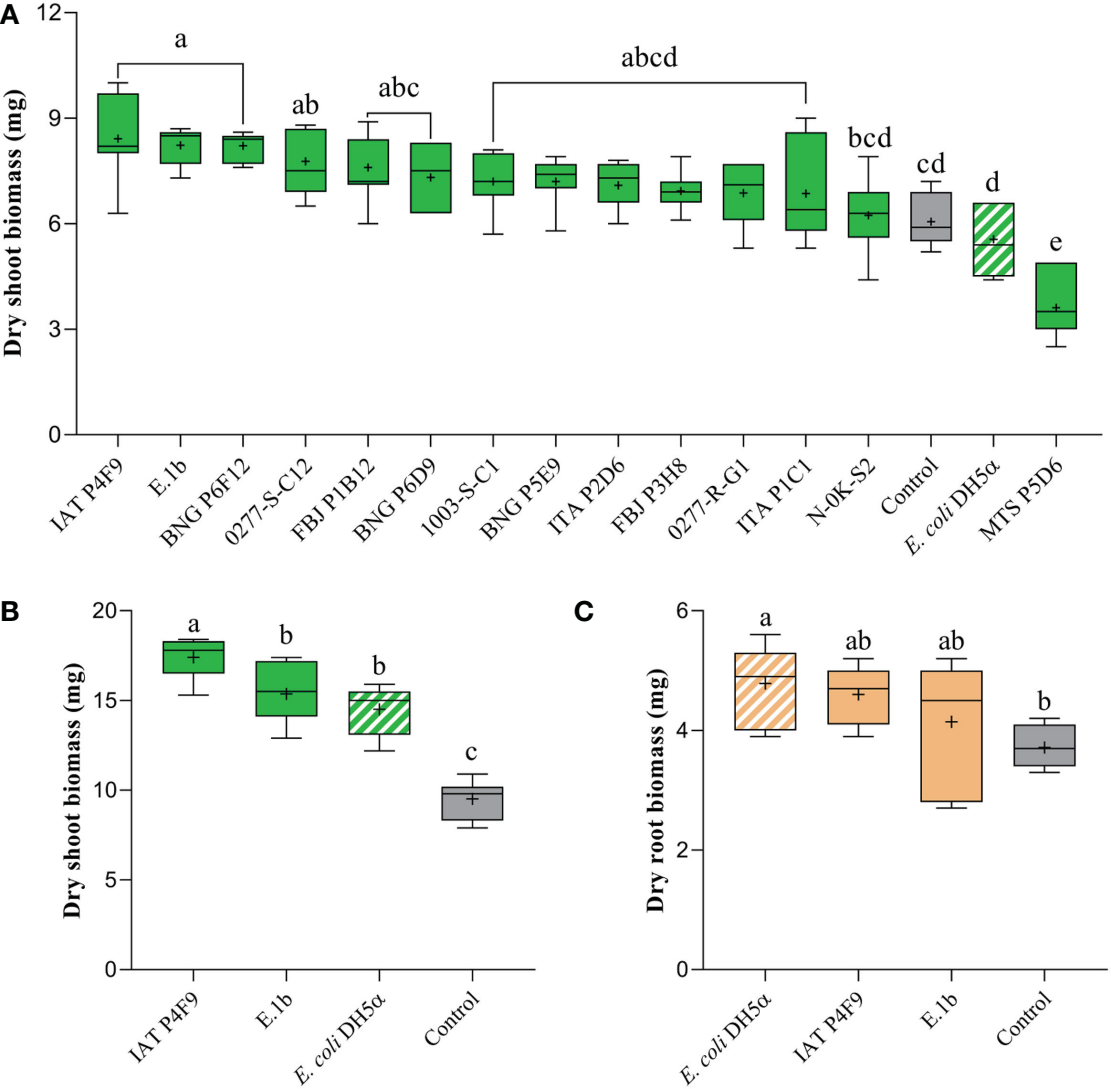
Effects of bacterial VOCs on **(A)** dry shoot biomass of rice plants, after 7 days of co-cultivation, and on **(B)** dry shoot and **(C)** root biomass, after 12 days of co-cultivation. Plants were co-cultivated with the bacterial isolates (full colored), the negative control *E. coli* DH5α (white striped), and grown without bacteria (control, gray). Significant differences (ANOVA followed by Tukey’s test, *p* < 0.05) among treatments are indicated by letters (*n* = 7).

Dry shoot biomass of rice co-cultivated for 7 days with the isolates IAT P4F9, E.1b, BNG P6F12, and 0277-S-C1 significantly increased by up to 39% (Tukey test, *p* < 0.05) when compared to plants grown in the absence of bacteria (control) and plants co-cultivated with *E. coli* DH5α (negative control) (**Figure 1A**). Contrastingly, rice seedlings co-cultivated with the isolate MTS P5D6 showed reduced dry shoot biomass by 40% (**Figure 1A**) and decreased root length, surface area, volume, and axial root length (**Supplementary Figure 3**).

Among the best isolates, IAT P4F9 and E.1b were selected to be evaluated in a 12-day co-cultivation assay. Plants co-cultivated with IAT P4F9 and E.1b had their dry shoot biomass significantly increased by 83% and 61%, respectively (**Figure 1B**). Curiously, plants co-cultivated with *E. coli* DH5α showed an increase in dry shoot (**Figure 1B**) and root biomass (**Figure 1C**) of 53% and 29%, respectively, and in the root morphological parameters (**Supplementary Figure 4**).

### 3.2 VOCs from E.1b and IAT P4F9 altered the rice metabolism of amino acids, citric cycle intermediates, carbohydrates, and lipids

To evaluate the effects of bacterial VOCs on plant metabolism, the isolate 1003-S-C1 was included as a negative control since VOCs from *E. coli* DH5α promoted rice growth after 12 days (**Figures 1B, C**). Two samples, one belonging to 1003-S-C1 and the other to E.1b treatments, were excluded from further analyses as they were confirmed to be outliers. A total of 41 non-volatile metabolites were identified (**Supplementary Table 2**). Fold change values from statistical analysis (*t*-test, *p* < 0.05) revealed that the concentrations of 33 metabolites were significantly different in at least one treatment comparison (**Supplementary Table 3**).

We performed HCA and PCA analyses to evaluate the general alterations on rice metabolomes due to VOCs (**Supplementary Figure 5**). On HCA, two main branches were formed separating the metabolome of plants co-cultivated with 1003-S-C1 from the others, and a sub-branch separated plants co-cultivated with IAT P4F9 and E.1b from control plants. PCA loadings (**Supplementary Table 4**) showed the metabolites that contributed most to the separation (e.g., 2-hydroxyisocaproate, 2-hydroxy-3-methylvalerate, lysine, 4-aminobutyrate, glutamine, asparagine, caprate, and tryptophan).

**Figure 2:**
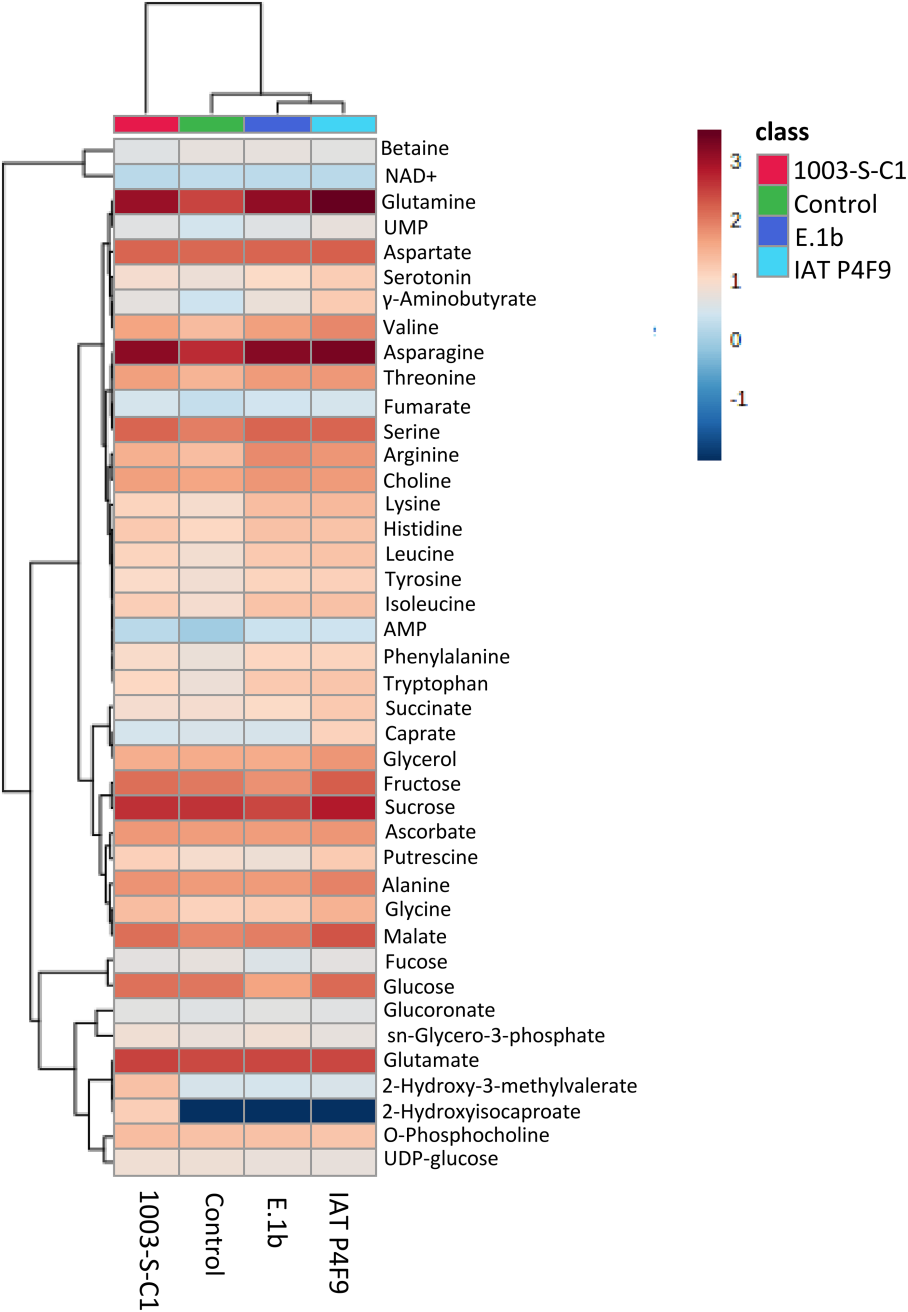
Heatmap of the metabolome of rice plants co-cultivated with the bacterial isolates (E.1b, IAT P4F9, and 1003-S-C1) and control plants (cultivated without bacteria). Columns represent each treatment and rows represent the different metabolites identified. The color code indicates the abundance of each metabolite (blue = low abundance; red = high abundance) based on the average of the Log_10_-transformed values (concentrations determined based on the reference signal of 0.5 mM TMSP-d_4_) (*n* = 4). The darkest blue indicates that the metabolite was not identified in plants subjected to the respective treatment.

The heatmap of metabolic data (**Figure 2**) showed that several amino acids were upregulated in the plants co-cultivated with the two promoter strains, compared with one or both controls (**Supplementary Table 3**). Arginine, asparagine, leucine, and lysine increased to a similar extent in rice co-cultivated with IAT P4F9 and E.1b, while the amino glutamine, glycine, and valine were significantly more abundant in IAT P4F9 compared with E.1b-treated plants. The sugars sucrose and the hexoses derived from its breakdown, glucose and fructose, besides fucose, displayed contrasting behavior in plants co-cultivated with both promoter strains: reduced in E.1b while increased or unchanged in IAT P4F9, compared with one or both controls. Rice grown with IAT P4F9 presented a higher abundance of the tricarboxylic acid (TCA) cycle intermediate malate and the lipid-related caprate and glycerol, compared with E.1b treatment.

Thus, 16 and 15 metabolic pathways had impact values above the threshold in plants co-cultivated with the isolates IAT P4F9 and E.1b, respectively, compared with the control (*p* < 0.05) (**Table 1**). Curiously, by comparing their metabolomes with the metabolome of the negative control, 13 and 5 pathways were identified, all commonly detected in the comparisons with the control plants (except the “glycerophospholipid metabolism” pathway). In agreement with the pairwise comparison, co-cultivation with both promoter strains affected the plant pathways “Arginine and proline metabolism”, “Arginine biosynthesis”, “Glycine serine and threonine metabolism”, “Glyoxylate and dicarboxylate metabolism”, and “Starch and sucrose metabolism”, compared with the control and the negative control. Interestingly, IAT P4F9, the strain that promoted the highest rice growth, also impacted “Alanine aspartate and glutamate metabolism”, “Butanoate metabolism”, “Citrate cycle (TCA cycle)”, “Pyrimidine metabolism”, “Pyruvate metabolism”, “Tryptophan metabolism”, and “Isoquinoline alkaloid biosynthesis”.

**Table 1:** Metabolic pathways affected in rice co-cultivated with IAT P4F9 and E.1b, compared to both controls.

Pathway		E.1b x CTL		E.1b x 1003		IAT x CTL		IAT x 1003	
		*p*-value	Impact value	*p*-value	Impact value	*p*-value	Impact value	*p*-value	Impact value
1	Alanine aspartate and glutamate metabolism	4.07E−05	0.78			4.26E−06	0.78	0.001786	0.78
2	Amino sugar and nucleotide sugar metabolism	0.013033	0.14						
**3**	**Arginine and proline metabolism**	**3.56E−05**	**0.26**	**0.009642**	**0.26**	**0.00014547**	**0.26**	**0.008907**	**0.26**
**4**	**Arginine biosynthesis**	**2.40E−06**	**0.17**	**0.00023**	**0.17**	**2.47E−06**	**0.17**	**0.002125**	**0.17**
5	Butanoate metabolism	9.33E−05	0.14			0.00026374	0.14	0.007963	0.14
6	Citrate cycle (TCA cycle)	0.037438	0.10			0.00065438	0.10	0.014002	0.10
7	Glutathione metabolism					0.00064237	0.12		
8	Glycerophospholipid metabolism							0.007264	0.11
**9**	**Glycine serine and threonine metabolism**	**9.51E−05**	**0.33**	**0.011322**	**0.33**	**3.08E−05**	**0.33**	**0.022301**	**0.33**
**10**	**Glyoxylate and dicarboxylate metabolism**	**9.85E−05**	**0.21**	**0.017527**	**0.21**	**1.52E−06**	**0.21**	**0.001638**	**0.21**
11	Isoquinoline alkaloid biosynthesis	0.000411	0.50			9.18E−05	0.50	0.025039	0.50
12	Nicotinate and nicotinamide metabolism					0.015218	0.22		
13	Phenylalanine metabolism	0.000754	0.47			0.0009957	0.47		
14	Pyrimidine metabolism	0.000438	0.13			1.58E−06	0.13	0.000108	0.13
15	Pyruvate metabolism	0.018797	0.15			0.00016627	0.15	0.013085	0.15
**16**	**Starch and sucrose metabolism**	**0.002559**	**0.16**	**0.022911**	**0.16**	**0.0035906**	**0.16**	**0.030246**	**0.16**
17	Tryptophan metabolism	4.27E−05	0.14			0.0001443	0.14	0.017431	0.14
18	Tyrosine metabolism	4.09E−05	0.22			0.00022762	0.22		

IAT, IAT P4F9; 1003, 1003-S-C1; CTL, Control (without bacteria).

Bold letters show commonly affected pathways.

### 3.3 *In vivo* validation of rice growth promotion mediated by bacterial VOCs

To evaluate if the growth promotion effects of VOCs produced by the isolates IAT P4F9 and E.1b were replicable *in vivo*, a new experiment was performed in a semi-open system where the plants were grown directly on the substrate. After 15 days, dry shoot biomass of plants co-cultivated with the isolate IAT P4F9 increased by 87% when compared to control plants, but no significant increase was observed in plants co-cultivated with isolates E.1b and 1003-S-C1 and with the strain *B. amyloliquefaciens* GB03 (**Supplementary Figure 6**).

### 3.4 Molecular identification of the best growth promoter isolates

The identification based on the 16S EzBiocloud database showed that the isolate IAT P4F9 presented 99.86% similarity with *Serratia marcescens* ATCC 13880(T), followed by *S. nematodiphila* DSM 21420(T) (99.72%), and *S. ureilytica* NiVa 51 (T) (98.39%). For E.1b, the highest similarity was with *Achromobacter insuavis* LMG 26845(T) (99.93%), followed by *A. ruhlandii* LMG 1866(T) (99.72%), and *A. aegrifaciens* LMG 26852(T) (99.72%). The best similarity hits to the bacterium isolate used as a negative control, 1003-S-C1, was with *Enterobacter cancerogenus* ATCC 33241(T) (99.93%), followed by *E. huaxiensis* 090008(T) (99.85%) and *E. bugandensis* EB-247(T) (99.78%). The best hit strains for all bacteria are presented in **Supplementary Table 5**, as well as the similarity information. In addition, the phylogenetic tree showed that IAT P4F9 is closest to *S. bockelmannii* and 1003-S-C1 is closest to *E. asburiae* (**Supplementary Figure 7**). Lastly, E.1b grouped with *Achromobacter* strains.

### 3.5 Volatilome profiles are species-specific

Volatilome profiling of the isolates IAT P4F9, E.1b, and 1003-S-C1, as well of *E. coli* DH5α (since this strain unexpectedly induced the plant growth at 12 days) revealed a total of 55 VOCs (**Supplementary Table 6**) classified into 17 chemical classes. The largest numbers of volatiles were produced by E.1b and IAT P4F9 isolates (37 and 28 compounds, respectively), whereas in the volatilome of *E. coli* DH5α, only eight VOCs were identified (**Supplementary Figure 8**). From the total VOCs detected, only four (3-methylbutan-1-ol, methanethiol, 2-phenylethanol, and nonan-2-one) were commonly identified among all volatilomes (**Supplementary Figure 8**). The growth promoter isolates exclusively produced 21 and 9 VOCs, respectively, and five VOCs were shared [heptan-2-one, (methyldisulfanyl)methane, undecane-2-one, toluene, and 1-(2-aminophenyl)ethanone]. The isolate 1003-S-C1 exclusively produced 3-methylbutyl acetate, 2-phenylacetaldehyde, hexadecanoic acid, and nonadecane.

The VOC profiles of the negative controls were more similar to each other than to the growth promoter isolates, and the E.1b volatilome was the most distinct among all (**Supplementary Figure 9**). PCA scores (**Supplementary Table 7**) established the VOCs that most explained the variation among isolates, such as phenylmethanol, (methyldisulfanyl)methane, (methyltrisulfanyl)methane, 2,4,6-trimethyl pyridine, anisole, methylsulfanylethane, phenol, butan-2-one, and undecan-2-one.

**Figure 3:**
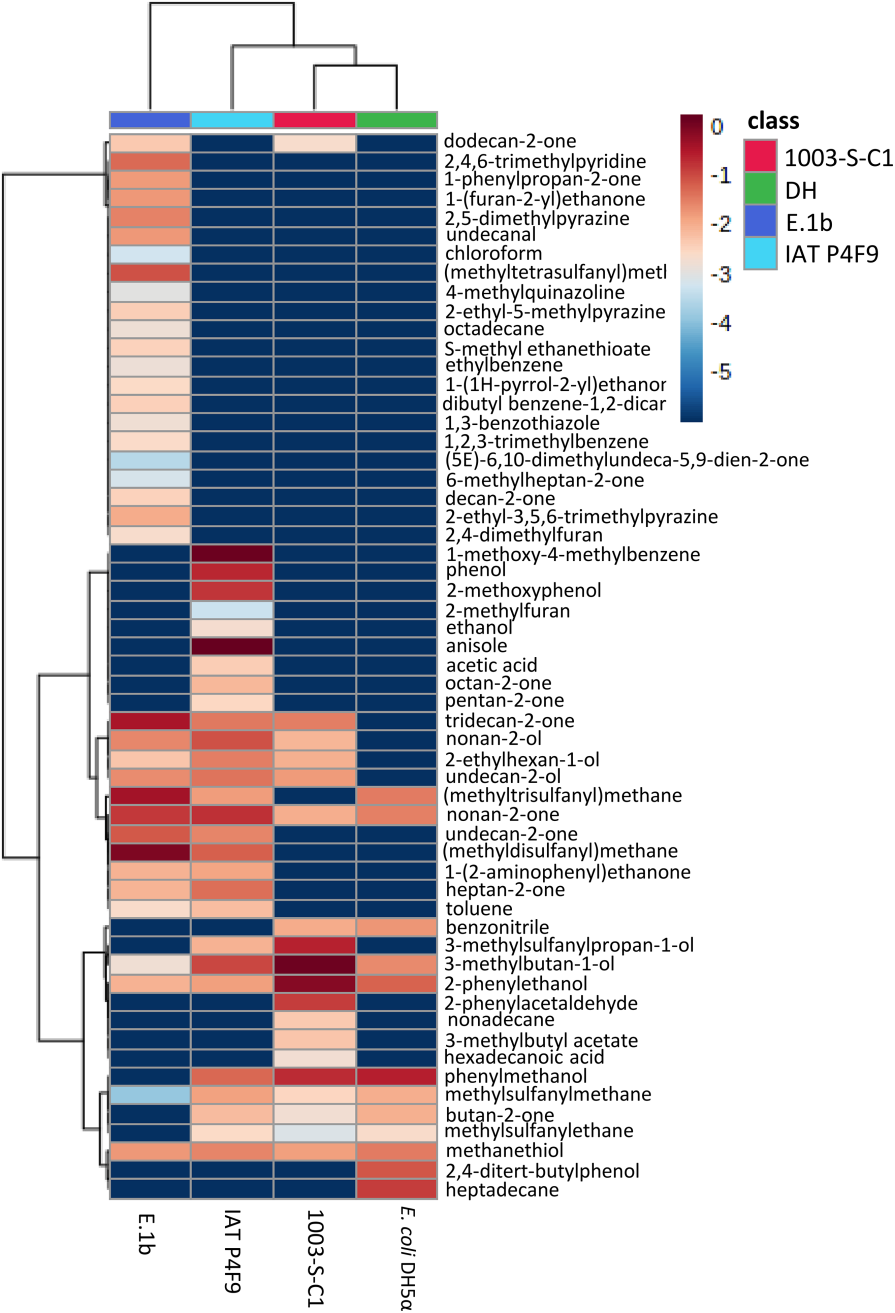
Heatmap of VOCs produced by the bacterial isolates (E.1b, IAT P4F9, and 1003-S-C1) and *E. coli* DH5α grown in LB medium. Columns represent each bacterium and rows represent the different VOCs detected. The color code indicates the abundance of each compound (blue = low abundance; red = high abundance), based on the average of the Log_10_-transformed values (peak areas were normalized by cis-3-Hexenyl acetate) (*n* = 5). The darkest blue indicates that the VOCs were not identified in the volatilome of the respective bacterium.

Although some VOCs were commonly produced by all bacteria, their abundance differed among them (**Figure 3** and **Supplementary Table 6**). For instance, nonan-2-one was produced in higher concentrations by the growth promoter isolates, while 2-phenylethanol was more abundant in the volatilomes of the negative controls (1003-S-C1 and *E. coli* DH5α).

### 3.6 *In vitro* validation of VOCs as growth promoters

The compound nonan-2-one (0.1 mg) induced a significant increase of 26% (Tukey test, *p* < 0.05) in dry shoot weight (**Supplementary Figure 10**) when compared to plants grown without synthetic VOCs (control). However, the compound (methyltrisulfanyl)methane [also known as dimethyl trisulfide (DMTS)] in the highest concentration caused a decrease in dry root weight by 41% when compared to control plants (**Supplementary Figure 10**). None of the other doses, compounds, and mixtures showed significant changes in shoot and root biomass (data no shown).

## 4 Discussion

### 4.1 Bacterial isolate VOCs promote rice growth

Plant growth promotion mediated by bacterial VOCs has been studied since 2003, when two *Bacillus* strains were demonstrated to promote *Arabidopsis* growth when cultivated on divided Petri dishes where only airborne signals could be exchanged between bacteria and plants (Ryu et al., 2003). After that, several other studies were performed showing the role of these volatile molecules as growth inducers (Bailly et al., 2014; Bitas et al., 2015; Piechulla et al., 2017; Tahir et al., 2017b; Jiang et al., 2019). Nevertheless, just a few studies were conducted with species of economic interest, especially monocots. To the best of our knowledge, no study was performed on rice so far. Our results showed that rice growth can be promoted by bacterial VOCs, with potential implications for agricultural food production.

Four of our bacterial isolates were capable of increasing plant dry shoot biomass up to 39% after 7 days of co-cultivation, when compared to plants cultivated without bacterium (control) (**Figure 1**). These results are in line with previous studies on other monocots showing that fresh shoot biomass of sorghum (*Sorghum bicolor*) was increased by 67% in co-cultivation with *Arthrobacter agilis* UMCV2 (Castulo-Rubio et al., 2015). Also, an 81% increase in total dry biomass of *Brachypodium distachyon* (L.) Beauv. induced by VOCs emitted by *B. amyloliquefaciens* GB03 was reported by Delaplace et al. (2015). As the induction of these phenotypes could be influenced by the exposure period to VOCs (Xie et al., 2009; Zou et al., 2010; Velázquez-Becerra et al., 2011), we re-evaluated the two isolates that promoted the highest rice growth, IAT P4F9 and E.1b, in a 12-day co-cultivation assay. Plants co-cultivated with both isolates had their dry shoot biomass augmented by 83% and 61%, respectively, in comparison to control plants, indicating that rice growth promotion increased over time. The first growth promotion effects (fresh shoot biomass) on *A. thaliana*, triggered by VOCs from *B. amyloliquefaciens* GB03, significantly started on the sixth day of co-cultivation and were observed until the 19th day, reaching an increase of nearly 260% (Zou et al., 2010). Therefore, it would be interesting to evaluate the long-term effect of these bacterial VOCs throughout the rice life cycle.

Curiously, the strain *E. coli* DH5α promoted rice plant growth after 12 days of co-cultivation. This strain was initially chosen as a negative control because it was not able to promote the growth of plants in previous studies (Ryu et al., 2003; Ryu et al., 2005; Kanchiswamy et al., 2015; Tahir et al., 2017b; Tahir et al., 2017c; Rath et al., 2018). However, Bailly et al. (2014) reported that VOCs emitted by *E. coli* DH5α induced an increase in biomass, secondary roots, and root hair length of *A. thaliana*. In the *Section 4.5*, we discuss which VOCs might be involved in rice growth promotion induced by this strain.

### 4.2 VOCs from IAT P4F9 and E.1b induce distinct metabolic changes in rice plants

By using ^1^H NMR, we characterized the metabolic profile of rice co-cultivated with the isolates and the control. The four metabolomes showed distinct metabolic profiles (**Figure 2**). Among the identified compounds, primary metabolites were the most abundant species, such as amino acids (44%), carbohydrates (15%), and organic acids (7%) (**Supplementary Table 2**). These compounds are the main intermediates of the plant’s central metabolism, including cellular respiration, energy demand, storage, and cell division (Fernie and Pichersky, 2015).

Amino acids are considered building blocks for other metabolites and cellular components, such as nucleotides, nitrogen compounds, chlorophylls, and proteins (Hildebrandt et al., 2015). Interestingly, changes in the concentration of up to 16 amino acids were observed in growth-promoted treatments when compared with the controls. Kang et al. (2015) showed that the abundance of 17 amino acids increased in cucumber treated with *Enterobacter* sp. SE992 inoculated in soil in comparison to control plants. In growing plants, the high demand for protein translation is provided by upregulating amino acid biosynthesis (Hildebrandt et al., 2015). The higher abundance of arginine, asparagine, leucine, and lysine in both promoter treatments compared to controls may denote common pathways by which rice growth is enhanced. Besides the classic role in protein synthesis, these aspartate-derived or related amino acids may act on glycolysis, starch regulation, lipid and nucleotide metabolism, defense response, photorespiration, and nitrogen metabolism (reviewed by Yang et al., 2020).

The efficient utilization of nitrogen considerably impacts plant biomass and yield (Yousaf et al., 2021). Arginine, asparagine, and glutamine are considered nitrogen carriers that store or transport this nutrient through plant vascular bundle (Miflin and Habash, 2002; Lea et al., 2007; Hildebrandt et al., 2015). The high levels of arginine, asparagine, and glutamine can indicate more efficient nitrogen assimilation and storage mediated by the promoter bacterial isolates, at a higher magnitude in IAT P4F9. However, further experiments are necessary to validate this hypothesis. Moreover, the higher abundance of specific amino acids in rice plants could be not only a consequence of improved translation but also an indication that other pathways regulating plant growth, such as nitrogen metabolism, plant immunity, and hormone synthesis, can be stimulated by VOCs.

Carbohydrates are the products of photosynthesis, serving as plant energy source and storage, as well as precursors of structural components for the cell walls (Flowers, 1981; Buchanan et al., 2015). Interestingly, the abundance of fructose, fucose, glucose, and sucrose differed in each treatment. The higher levels of these sugars, especially sucrose, might suggest that photosynthesis was upregulated in plants co-cultivated with IAT P4F9, while the mechanism induced by E.1b seems to be less clear, as this isolate displayed an opposite effect on sugars. Such results may indicate that carbohydrate metabolism plays an important role in the molecular responses resulting from rice growth promotion induced by the bacterial VOCs.

The TCA intermediates malate, succinate, and fumarate were up to 1.52, 1.19, and 0.60-fold more abundant, respectively, in rice co-cultivated with IAT P4F9 compared to control and/or negative control plants. These organic acids are derived from the oxidation of carbohydrates produced by photosynthesis and participate in several metabolic pathways, such as respiration/energy metabolism, catabolism of amino acids, and even plant tolerance to stresses (Abadía et al., 2002; Zell et al., 2010; Hildebrandt et al., 2015). Interestingly, “citrate cycle” and “pyruvate metabolism” were also pointed out as affected pathways in plants co-cultivated with both promoter strains. Together, the levels of sucrose and TCA intermediates show that the process of transforming sugars into energy might be more active when rice was co-cultivated with IAT P4F9. Moreover, the higher amino acid contents in these plants might be an indicator of augmented translation, which could ultimately lead to higher growth and biomass.

Our study showed for the first time that microbial VOCs induce distinct metabolic changes in rice. Although the metabolic profiles cannot solely explain the molecular responses involved in growth promotion, they shed a light on possible pathways and targets for further investigations. Moreover, other approaches (e.g., transcriptomics, proteomics, and mutant evaluation) are needed to elucidate the molecular and metabolic mechanisms involved in bacterial VOC-mediated plant growth.

### 4.3 VOCs from IAT P4F9 also promotes rice growth *in vivo*

Although the *in vitro* co-cultivation system is a practical method to screen bacteria as potential PGPB, the bacterial VOCs might be concentrated in the Petri dishes generating a response that would not occur in a natural environment. Thus, the isolates IAT P4F9 and E.1b were also evaluated in a semi-open system adapted from Park et al. (2015). Surprisingly, only VOCs emitted by IAT P4F9 promoted the growth of rice plants, which showed an 87% increase in shoot dry biomass. This was similar to the results from the *in vitro* experiment results (83% increase in shoot dry biomass in plants co-cultivated with IAT P4F9 compared to control plants). Although E.1b VOCs resulted in a 61% increase *in vitro*, this isolate did not affect plant growth *in vivo*. In the semi-open system used by Park et al. (2015), tobacco plants (*N. tabacum* cv. Xanthi-nc) co-cultivated with *P. fluorescens* SS101 had an increase of 150% in fresh biomass compared to control plants. Furthermore, an increase of 198% in dry biomass was observed in tomato plants co-cultivated with *Bacillus subtilis* SYST2 for 30 days *in vivo* (Tahir et al., 2017b). As for cucumber plants (*Cucumis sativa* L. cv backdadagi) cultivated in a greenhouse, their fresh shoot and root biomass increased by 57% and 30%, respectively, after 14 days in contact with VOCs emitted by *B. amyloliquefaciens* GB03 (Song et al., 2019). VOCs from *B. amyloliquefaciens* GB03 had no effect on rice plants in our experiment *in vivo*, strengthening the specificity of the interaction between plant and bacteria. Our results demonstrate for the first time that VOCs can promote growth of a monocot plant in semi-open systems. This reinforces their potential for field use, although it does not eliminate the need for further investigations on the long-term effects of bacterial volatiles on rice growing in paddy soil, approaching the ability of VOCs to diffuse into the water.

### 4.4 Bacterial isolates belong to different genera

Since we observed that the isolates IAT P4F9 and E.1b efficiently promoted rice growth, we investigated the taxonomic assignment of these strains by sequencing the full-length 16S rRNA and performed phylogenetic analysis using the 16S rRNA and *rpoB* nucleotide sequences for IAT P4F9 and the 16S rRNA sequence for the E.1b strain. According to EzBiocloud, the high identity of IAT P4F9 was with *S. marcescens* ATCC 13880(T) (98.86%). Phylogenetic analysis also supports the taxonomic assignment to *Serratia* genus, a γ-proteobacterium. Previous studies have reported antagonistic effects on *A. thaliana* growth caused by *Serratia* species (Kai et al., 2008; Blom et al., 2011a; Blom et al., 2011b; Heenan-Daly et al., 2021; Plyuta et al., 2021). Blom et al. (2011a) showed that *S. marcescens, S. entomophilia*, and *S. proteamaculans* were able to promote *A. thaliana* growth, but *S. plymuthica* could trigger positive or negative effects. In addition, Plyuta et al. (2021) showed that *S. proteamaculans* and *S. plymuthica* strongly inhibited *A. thaliana* growth.

The 16S of the isolate E.1b showed the highest identity with *Achromobacter insuavis* LMG 26845(T) (99.93%), and phylogenetics analysis reinforces its identification. Several studies report species of *Achromobacter* (β-Proteobacteria) acting as plant growth-promoting bacteria (Mayak et al., 2004; Jha and Kumar, 2009; Abdel-Rahman et al., 2017; Wang et al., 2020; Kapadia et al., 2022), but only a few have focused on VOC mechanisms. Recently, VOCs from *Achromobacter* sp. 5B1 were shown to increase both shoot and root biomass of *A. thaliana* by 66% and 55%, respectively (Jiménez-Vázquez et al., 2020), while no alteration was observed in lettuce (*Lactuca sativa*) co-cultivated with a strain from the same genus (Minerdi et al., 2011). Such opposite phenotypes can occur because VOC-mediated growth promotion is species-specific. The phenotype depends on the species or strain (bacterium and plant) that are interacting, the culture medium, growth condition, and other factors. For instance, increased dry biomass of *Mentha piperita* co-cultivated with *Pseudomonas fluorescens* WCS417r (Santoro et al., 2011) and tobacco (*Nicotiana tabacum* cv. Xanthi-nc) co-cultivated with *P. fluorescens* SS101 (Park et al., 2015) contrasts with reduced primary root formation in *A. thaliana* co-cultivated with *P. fluorescens* CHAO, probably due to hydrogen cyanide emission (Rudrappa et al., 2008). Thus, these results reinforce the importance of γ- and β-Proteobacteria for plant productivity and soil health, as previous studies have reported that they help in nutrient acquisition and provide plant protection (Lugtenberg and Kamilova, 2009; Malla et al., 2022).

### 4.5 Volatilome analysis revealed compounds known to promote plant growth

Bacterial volatilomes are diversified and dynamic, i.e., besides the differences in VOC profiles among bacteria, VOC production may change in quantity and quality due to different factors, such as media composition and growth period (Macías-Rubalcava et al., 2018; Heenan-Daly et al., 2021; Freitas et al., 2022). To characterize the volatilome produced by our bacterial isolates and further validate which VOCs are the bioactive compounds that induce growth, VOCs emitted by both promoter isolates IAT P4F9 and E.1b, and the isolate 1003-S-C1 (non-promoter, negative control) were analyzed by HS-SPME/GC-MS. We also evaluated the *E. coli* DH5α volatilome to investigate why this strain promoted rice growth after 12 days of co-cultivation. All these bacteria belong to different genera, and in total, 58 VOCs were identified. These compounds can be classified according to the microbial volatile organic compounds database (mVOC, Lemfack et al., 2018) into several chemical classes, which are mostly benzenoids (24%), ketones (18%), sulfides (11%), alcohols (9%), furans (5%), pyrazines (5%), alkanes (4%), and others (24%). These are the most frequent chemical classes found in bacterial volatilomes (Wenke et al., 2012; Peñuelas et al., 2014).

Although it was not the focus of our study, we have also identified DMTS as a growth-inhibitor bio compound, at last in the highest concentration evaluated (**Supplementary Figure 10**). This compound is reported as capable of inhibiting the growth of several microorganisms (Ossowicki et al., 2017; Agisha et al., 2019; Guevara-Avendaño et al., 2019; Freitas et al., 2022) and inducing the expression of defense-related genes in apple fruit (Sun et al., 2022); however, it remains unclear if this could be due to a cytotoxic effect as our data suggested.

Here, we evaluated six compounds, individually and mixed in solutions, of which nonan-2-one (0.1 mg) increased dry shoot weight *in vitro* assays by up to 26% (**Supplementary Figure 10**). This compound was one of the five VOCs commonly identified in all the characterized volatilomes. Nonetheless, IAT P4F9 and E.1b produced nonan-2-one in higher concentrations compared with the negative controls. Fincheira et al. (2017) previously showed that nonan-2-one, identified in the volatilome of *Bacillus* sp. BCT9, was able to increase lettuce biomass by up to 48% at doses of 0.001 mg after 10 days of exposure to the volatile.

Although other compounds identified in the volatilome of our bacterial isolates were not evaluated or did not present significant effects on rice growth, they were validated in other plants. For instance, the application of 1.0 mg of DMDS promoted tobacco growth (Meldau et al., 2013), while 0.471 and 0.0471 mg of DMDS reduced the growth of *A. thaliana* by 20% and 30%, respectively (Kai et al., 2010). A VOC with a similar structure and belonging to the same chemical class (sulfide), DMTS, induced *A. thaliana* growth (Cordovez et al., 2018). Park et al. (2015) showed a significant increase in the fresh weight of *N. tabacum* when treated with butan-2-one. Camarena-Pozos et al. (2019) reported that 2-phenylethanol induced *A. thaliana* and *Agave tequilana* growth, while 3-methylbutan-1-ol induced *A. thaliana* and *A. salmiana* growth. Besides nonan-2-one, tridecan-2-one and undecane-2-one (both at 10^−6^ mg) have also elicited an increase in lettuce dry weight by 23% and 25%, respectively (Fincheira et al., 2017). Although a given compound can cause different effects on distinct plant species, we can hypothesize that one or some of these compounds might be responsible for the growth promotion of rice. Two of them (undecane-2-one and DMDS) were found exclusively on the volatilomes of the growth promoter isolates IAT P4F9 and E.1b. Intriguingly, 1003-S-C1 also shares some bioactive compounds that promote plant growth, such as 3-methylbutan-1-ol, 2-phenylethanol, and nonan-2-one, reinforcing that the growth-promoting phenotype is reached through a junction of several parameters, not only the presence or absence of a given compound.

Regarding growth promotion of rice co-cultivated with *E. coli* DH5α, some VOCs already validated as growth inducers were found in the volatilome of this strain. As already discussed in *Section 4.1*, Bailly et al. (2014) showed that *E. coli* DH5α can promote plant growth; the authors attributed these effects to the emission of VOC 1H-indole. This compound was not present in *E. coli* DH5α volatilome (**Figure 3**) because, after the manual peak-to-peak curing, it was considered peaks from single VOCs (i.e., co-elution metabolites were discarded) and VOCs present in at least three biological replicates and with an area at least twice as large in the samples as in the control (only LB media). However, as shown in **Supplementary Figure 11**, this compound was produced by this strain, but it co-eluted with others. To validate these data, we reanalyzed the *E. coli* DH5α volatilome using a quadrupole GC/MS and the 1H-indole eluted in a distinct GC peak (**Supplementary Figure 12**). In addition, as previously mentioned, other compounds that might have a growth-inducing effect were found in *E. coli* DH5α volatilome, such as nonan-2-one (Fincheira et al., 2017), butan-2-one (Park et al., 2015), 3-methyl-1-butanol, and 2-phenylethanol (Camarena-Pozos et al., 2019).

In conclusion, our study identified two bacterial isolates, belonging to *Achromobacter* and *Serratia* genera, which produced VOCs capable of inducing rice growth *in vitro*. Among these VOCs, nonan-2-one was validated *in vitro* as a growth inducer compound in this species. The bacterial VOCs also induced metabolic changes in these plants, especially in primary metabolites. In particular, rice energy metabolism seems to be upregulated by the isolate IAT P4F9. However, further studies are necessary to better understand the molecular changes mediated by bacterial VOCs on rice. Finally, this study also shows, for the first time, the *in vivo* growth promotion of a monocot, which is a potential sustainable tool for increasing rice productivity in the field.

## Data availability statement

The original contributions presented in the study are publicly available. This data can be found here: https://www.ncbi.nlm.nih.gov/, accession numbers OP186019, OP186020, OP186021, OP893533 and OP893532.

## Author contributions

NA and OA carried out the experiments and performed the data analyses. OA and JO wrote the first draft of the manuscript. AM and GP contributed to investigation. MS, GP and MC-R contributed to data analysis and wrote sections of the manuscript. JO contributed to conception, design of study, supervision, and funding acquisition. All authors revised the final manuscript. All authors contributed to the article and approved the submitted version.
