# Supplementary figures and images for: Bacterial volatile organic compounds (VOCs) promote growth and induce metabolic changes in rice
Source: Front Plant Sci. 2023 Feb 9;13:1056082. doi: 10.3389/fpls.2022.1056082 (PMC9948655; doi:10.3389/fpls.2022.1056082)

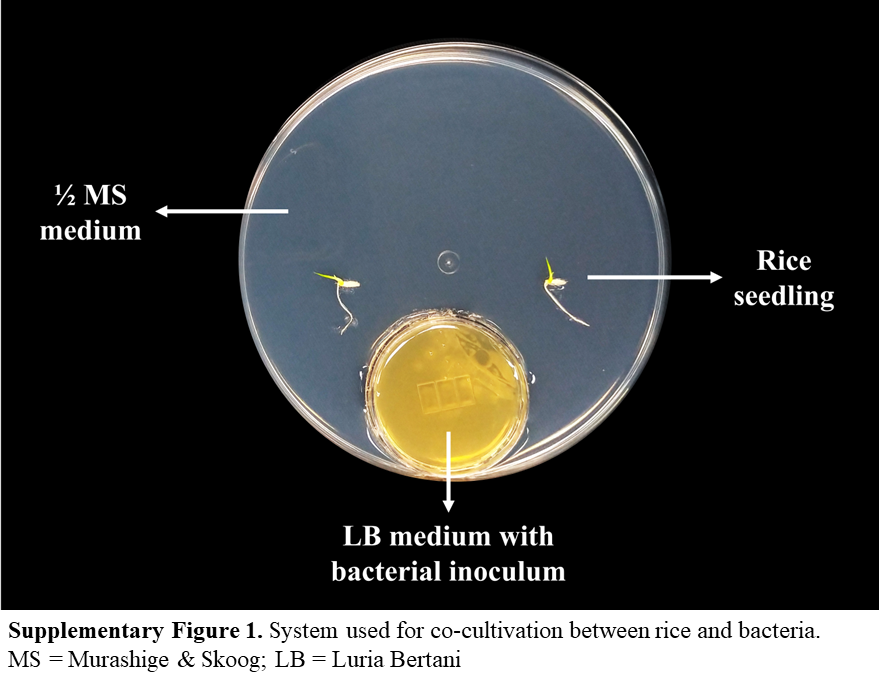

Supplement: Supplementary file 1 [file Image_1.tif]

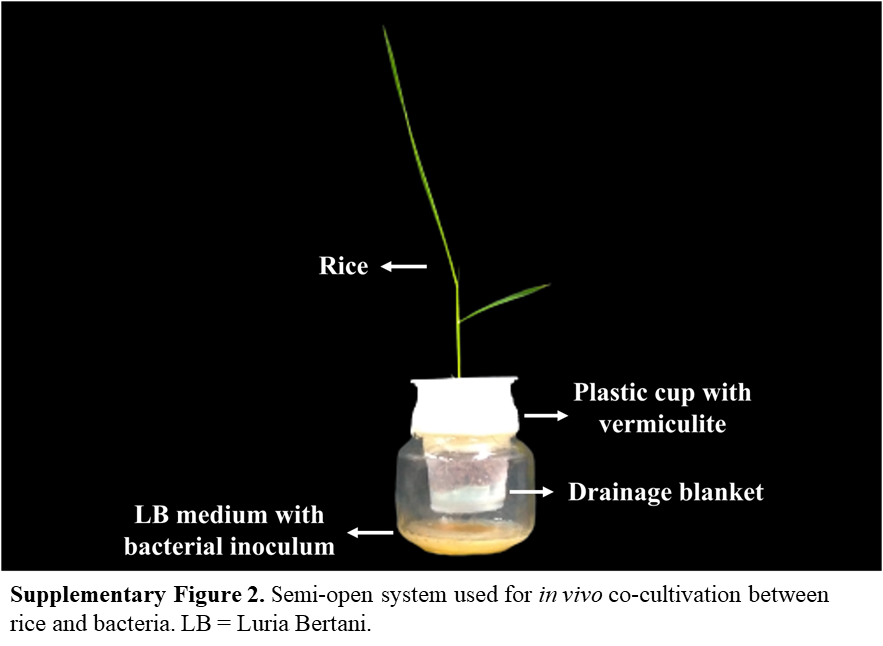

Supplement: Supplementary file 2 [file Image_2.tif]

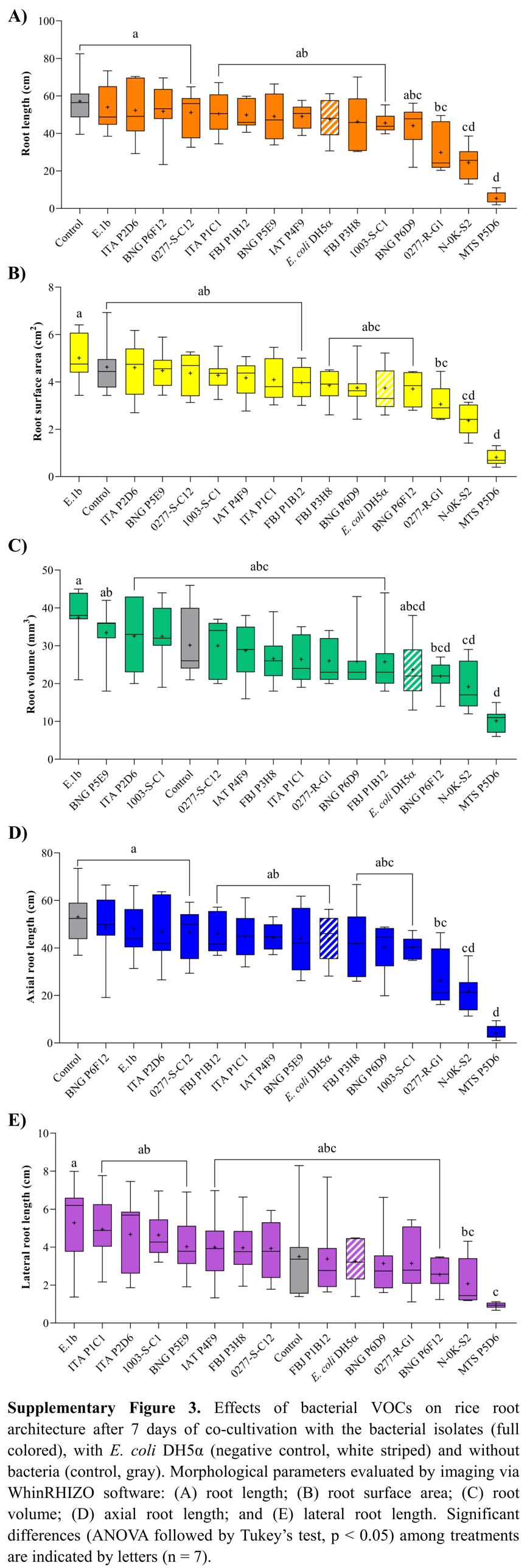

Supplement: Supplementary file 3 [file Image_3.tif]

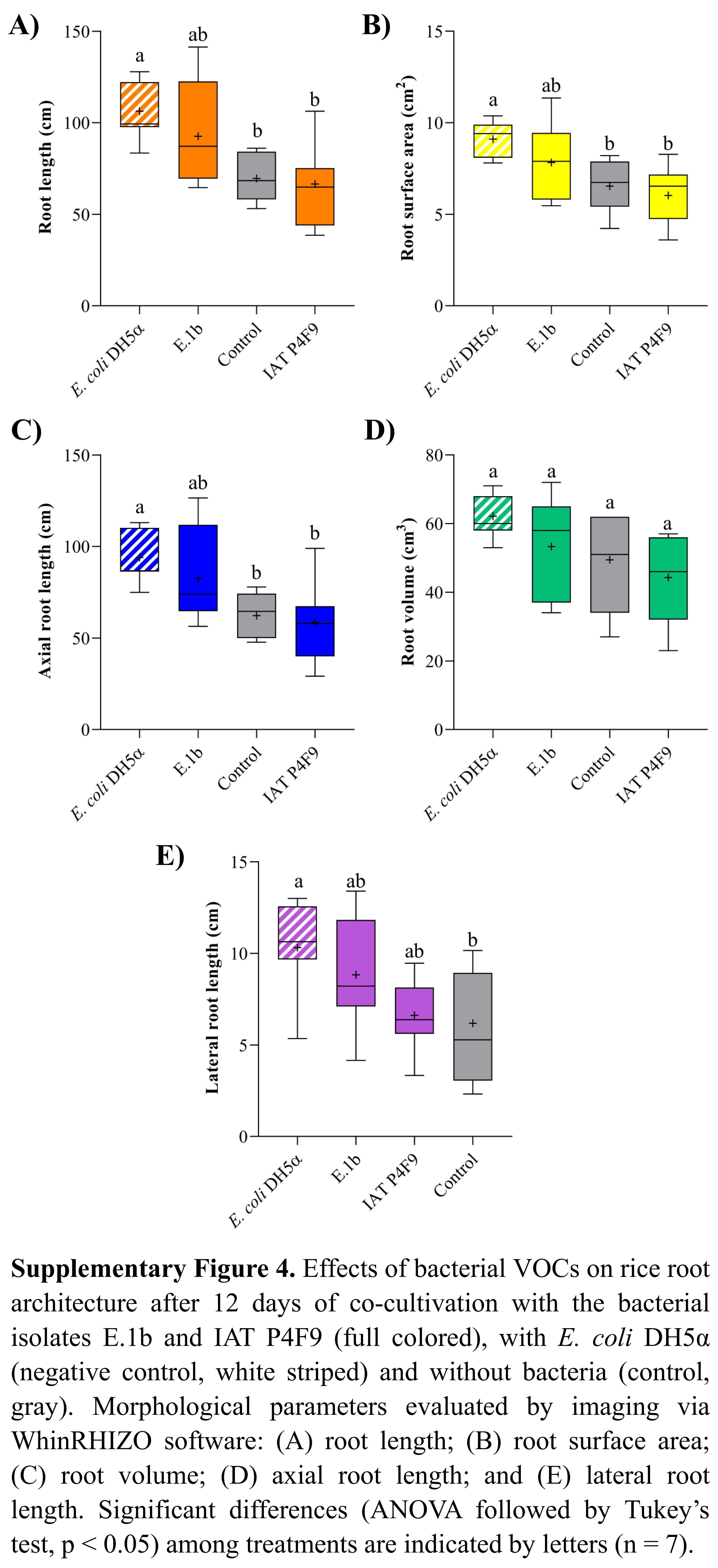

Supplement: Supplementary file 4 [file Image_4.tif]

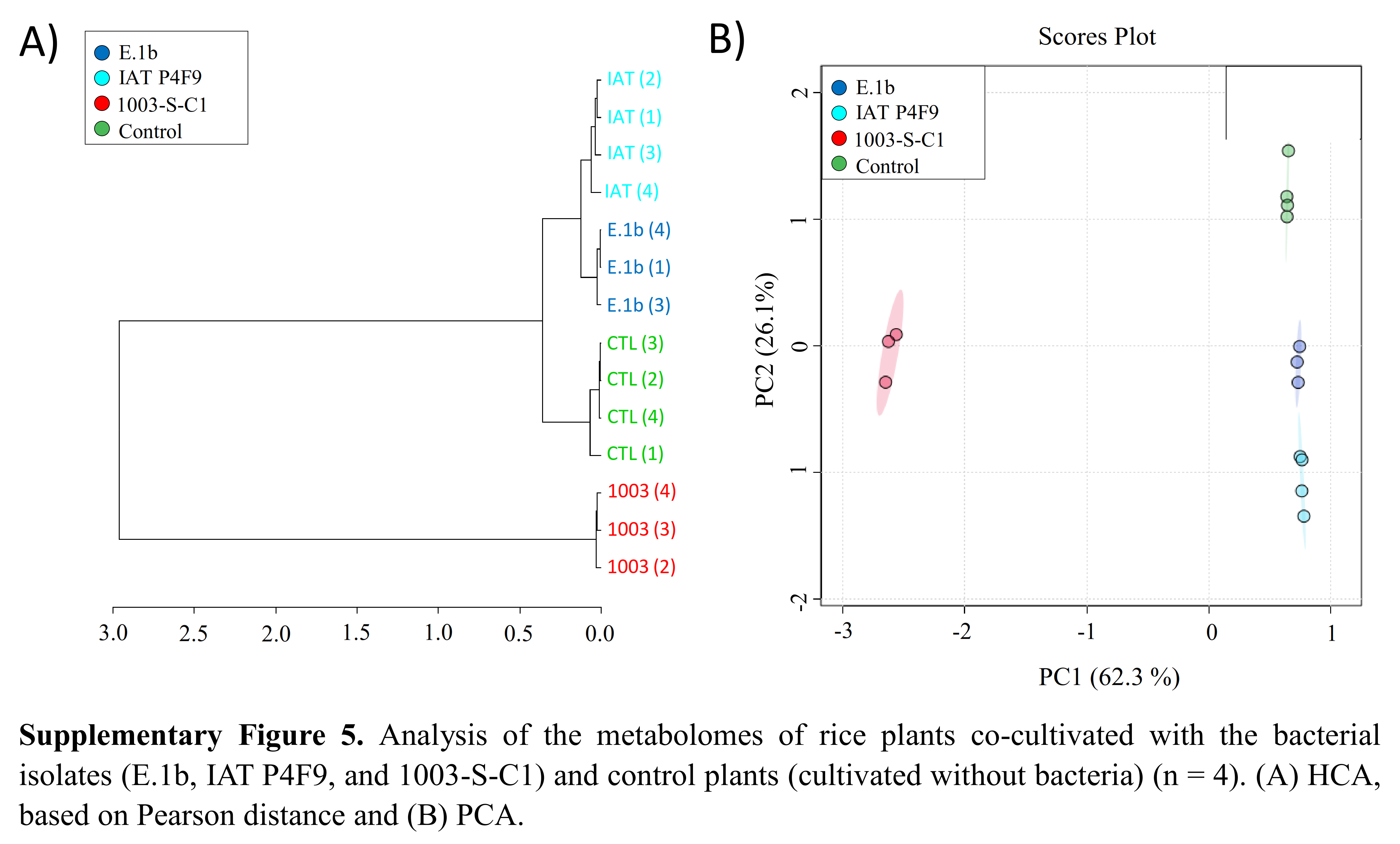

Supplement: Supplementary file 5 [file Image_5.tif]

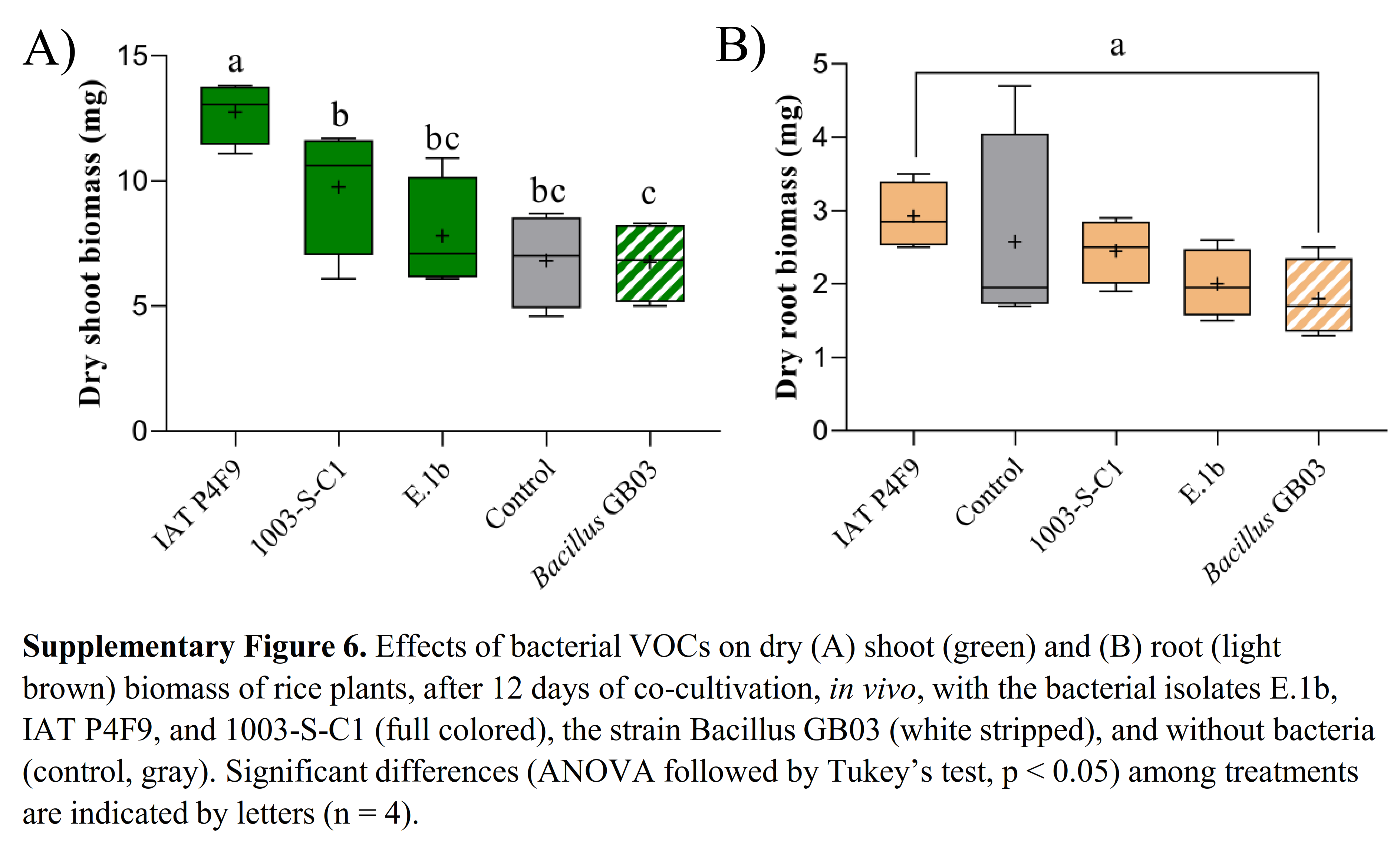

Supplement: Supplementary file 6 [file Image_6.tif]

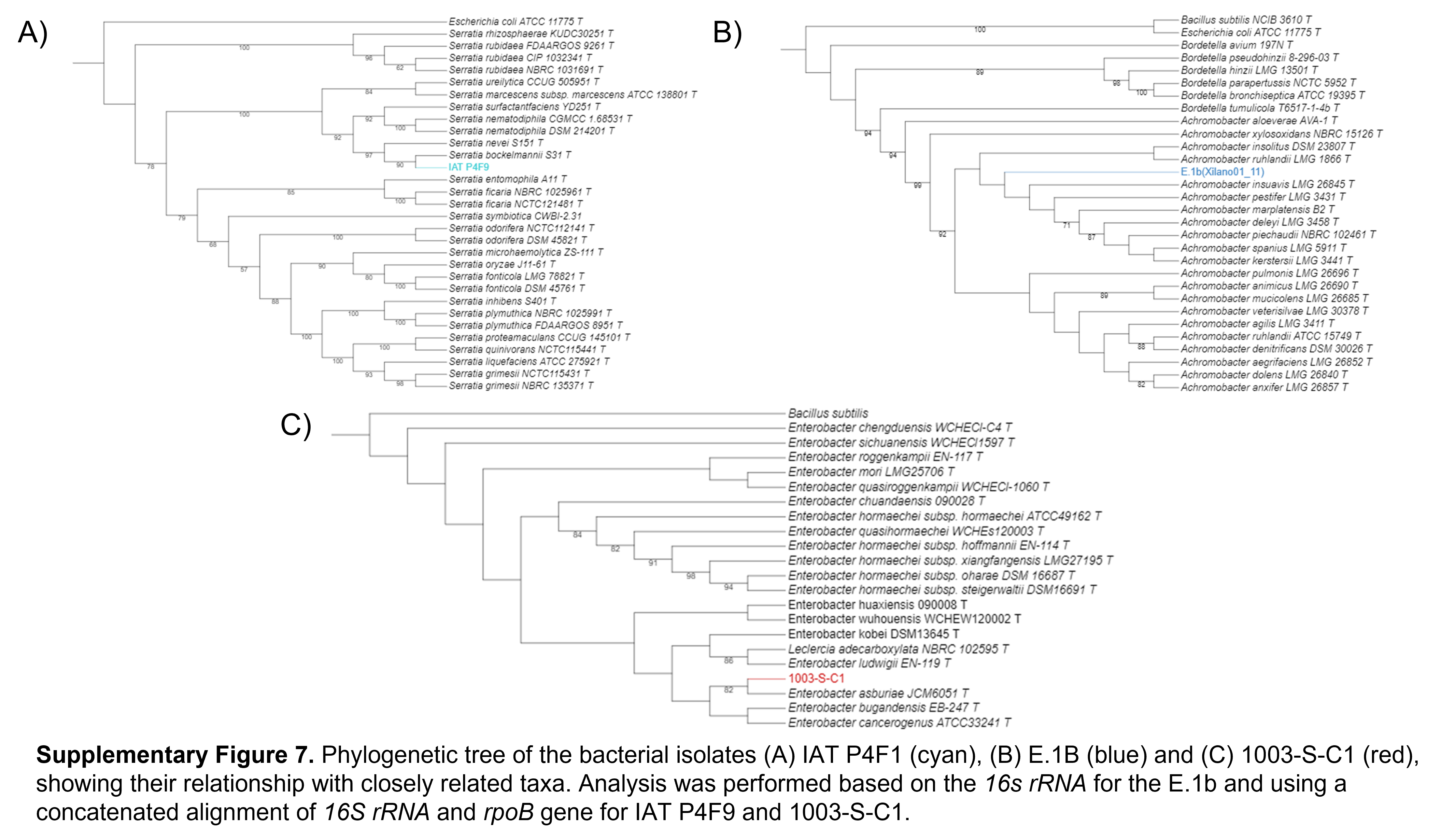

Supplement: Supplementary file 7 [file Image_7.tif]

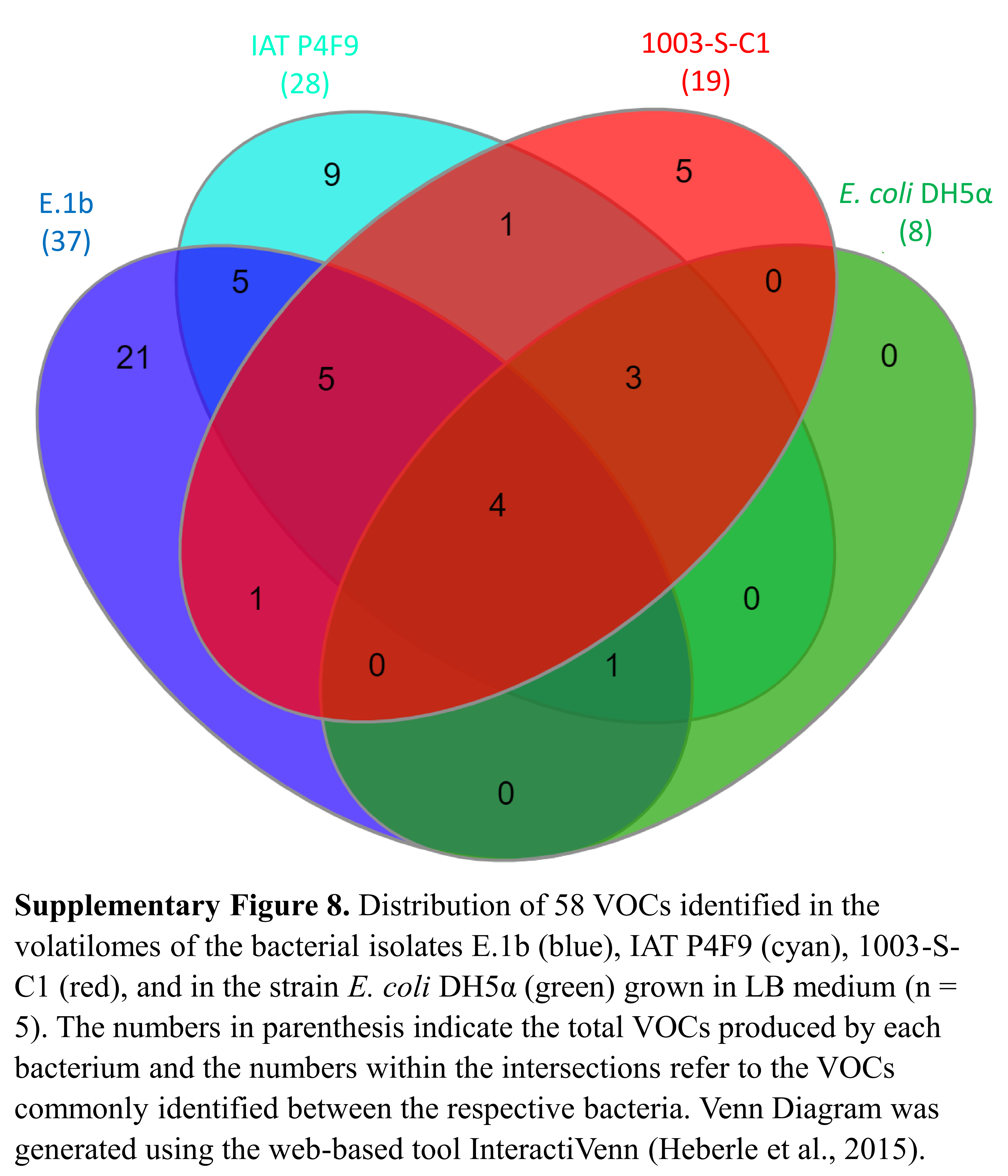

Supplement: Supplementary file 8 [file Image_8.tif]

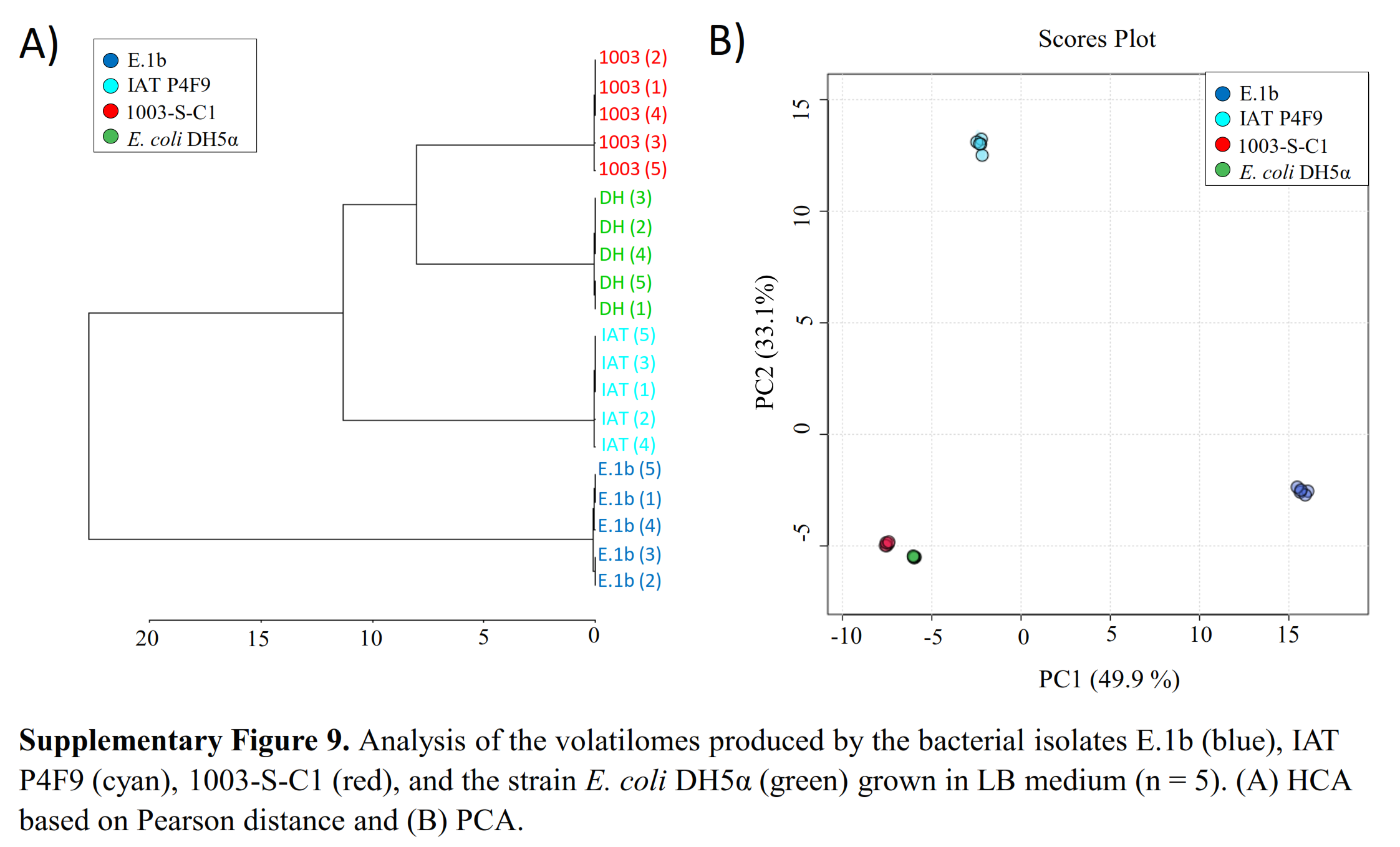

Supplement: Supplementary file 9 [file Image_9.tif]

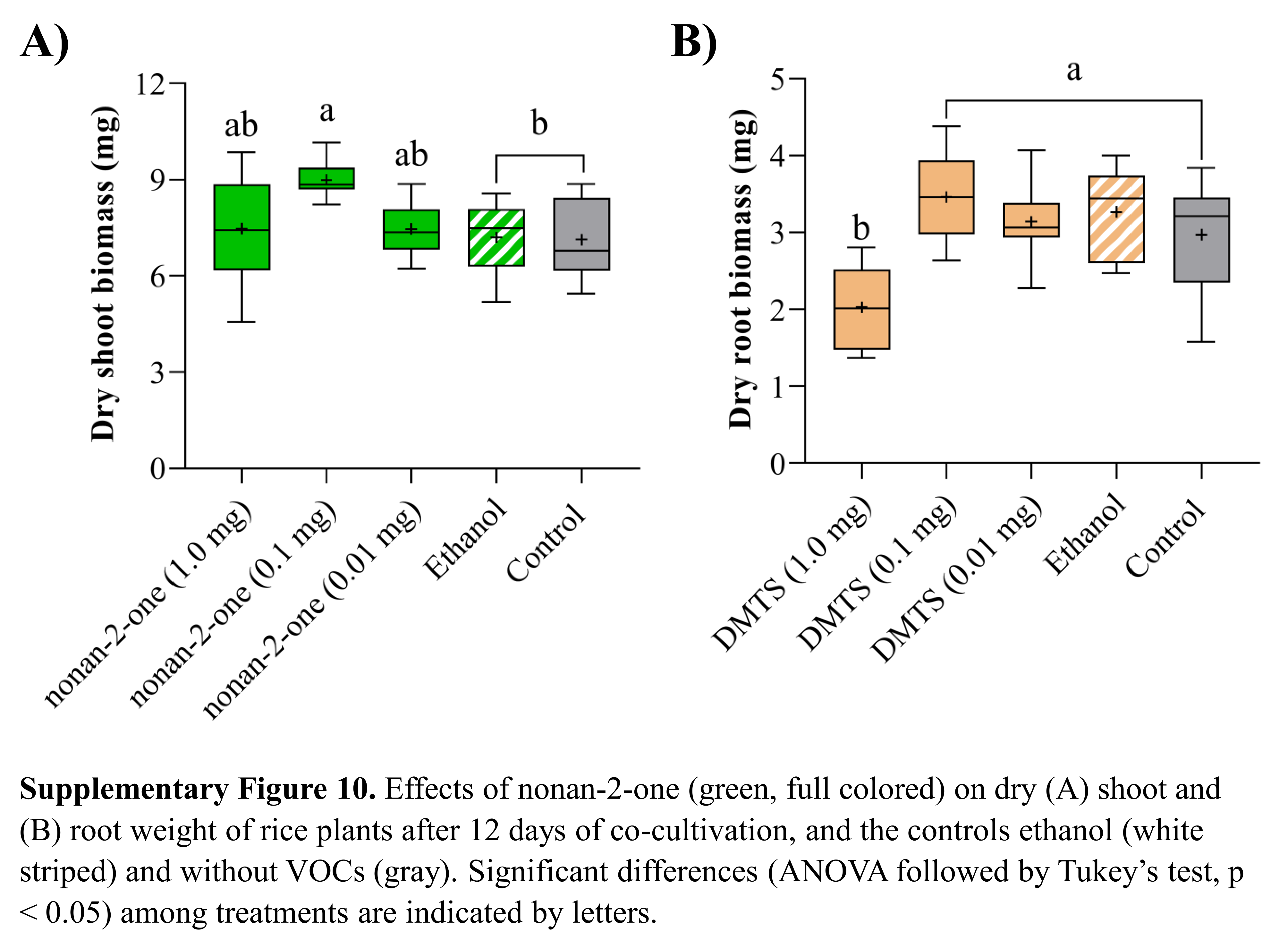

Supplement: Supplementary file 10 [file Image_10.tif]
