# Supplementary material for: Bacterial volatile organic compounds (VOCs) promote growth and induce metabolic changes in rice
Source: Front Plant Sci. 2023 Feb 9;13:1056082. doi: 10.3389/fpls.2022.1056082 (PMC9948655; doi:10.3389/fpls.2022.1056082)
Supplement: Supplementary file 13 [file Table_1.docx]

Supplementary Material

The bacterial strains evaluated in this work were isolated from sugarcane and energy cane fields (rhizospheric soil and root), as well as from composting of filter cake. 1,6 g of rhizospheric soil from different Brazilian´s states were serial diluted in sterile water (10-fold dilution), and the suspensions were plated in LB medium (Bertani, 1951). For isolation of endophytic bacteria, 1 g of roots superficially sterilized was crushed in saline solution (3,4 g.L^-1^ KH_2_PO_4_; 0,2 g.L^-1^ MgSO_4_.7H_2_O; 0,1 g.L^-1^ NaCl; 0,02 g.L^-1^ CaCl_2_.2H_2_O; 4,5 g.L^-1^ KOH) and the suspension was serial diluted and plated in LB medium. 5 g of composting material were diluted in sterile water, serial diluted and plated in minimum media [50 mL.L^-1^ 20X saline solution (6 g.L^-1^ NaNo_3_, 1,5 g.L^-1^ KH_2_PO_4_, 0,5 g.L^-1^ KCl, 0,5 g.L^-1^ MgSO_4_), 200 µL.L^-1^ trace elements solution (10 g.L^-1^ EDTA, 4,4 g.L^-1^ ZnSo_4_.7H_2_O, 1g.L^-1^ MnCl_2_.4H_2_O, 0,32 g.L^-1^ CoCl_2_.6H_2_O, 0,315 g.L^-1^ CuSO_4_.5H_2_O, 0,22 g.L^-1^ (NH_4_)6Mo_7_O_24_.4H_2_O, 1,47 g.L^-1^ CaCl_2_.2H_2_O, 1 g.L^-1^ FeSO_4_.7H_2_O), 15 g.L^-1^ agar] supplemented with different carbon sources (1% of xylan, xylose, carboxy methyl cellulose or glucose). The isolated microorganisms were identified by sequencing of the 16S rRNA V3-V5 region (The Human Microbiome Project Consortium, 2012) and stored at -80 ºC in 40% glycerol.

**Supplementary Table 1.** Bacterial isolates selected for the rice co-cultivation assays and their identification.

| **Name** | **Genus** | **Origin** | **Brazilian state** |
| --- | --- | --- | --- |
| BNG P6D9 | *Citrobacter* sp. | Sugarcane soil | Tocantins |
| FBJ P1B12 | *Pseudomonas* sp. | Sugarcane soil | São Paulo |
| BNG P5E9 | *Kosakonia* sp. | Sugarcane root | Tocantins |
| BNG P6F12 | *-* | Sugarcane root | Tocantins |
| IAT P4F9 | *Serratia* sp. | Sugarcane root | Mato Grosso |
| ITA P1C1 | *Kosakonia* sp. | Sugarcane root | Mato Grosso |
| ITA P2D6 | *Enterobacter* sp. | Sugarcane root | Mato Grosso |
| FBJ P3H5 | *Pantoea* sp. | Sugarcane root | São Paulo |
| MTS P5D6 | *Pseudomonas* sp. | Sugarcane soil | São Paulo |
| E.1b(Xilano01_11) | *Achromobacter* sp. | Composting of filter cake | São Paulo |
| 1003-S-C1 | *Enterobacter* sp. | Sugarcane soil | São Paulo |
| 0277-R-G1 | *Bacillus* sp. | Sugarcane root | São Paulo |
| 0277-S-C12 | *Enterobacter* sp. | Sugarcane soil | São Paulo |
| N-0K-S2 | *-* | Energy cane soil | São Paulo |

**REFERENCES**

Bertani, G. (1951). Studies on lysogenesis. I. The mode of phage liberation by lysogenic Escherichia coli. *J. Bacteriol.* 62, 293–300. doi:10.1128/JB.62.3.293-300.1951.

The Human Microbiome Project Consortium (2012). Structure, function and diversity of the healthy human microbiome. *Nature* 486, 207. doi:10.1038/NATURE11234.
