# Supplementary material for: Bacterial volatile organic compounds (VOCs) promote growth and induce metabolic changes in rice
Source: Front Plant Sci. 2023 Feb 9;13:1056082. doi: 10.3389/fpls.2022.1056082 (PMC9948655; doi:10.3389/fpls.2022.1056082)
Supplement: Supplementary file 14 [file Table_2.docx]

Supplementary Material

**Supplementary Table 2.** Metabolites identified in shoots of rice co-cultivated with the bacterial isolates E.1b, IAT P4F9 and 1003-S-C1 and control plants.

| **Metabolite** | **KEGG** | **Formula** | **Classification** |
| --- | --- | --- | --- |
| 2-Hydroxy-3-methylvalerate | - | C_6_H_12_O_3_ | Hydroxy fatty acids |
| 2-Hydroxyisocaproate | C03264 | C_6_H_12_O_3_ | Hydroxy fatty acids |
| Alanine | C00041 | C_3_H_7_NO_2_ | Amino acids |
| AMP | C00020 | C_10_H_14_N_5_O_7_P | Nucleic acids |
| Arginine | C00062 | C_6_H_14_N_4_O_2_ | Amino acids |
| Ascorbate | C00072 | C_6_H_8_O_6_ | Vitamins |
| Asparagine | C00152 | C_4_H_8_N_2_O_3_ | Amino acids |
| Aspartate | C00049 | C_4_H_7_NO_4_ | Amino acids |
| Betaine | C00719 | C_5_H_11_NO_2_ | Amino acids |
| Caprate | C01571 | C_10_H_19_O_2_ | Fatty acids |
| Choline | C00114 | C_5_H_14_NO | Vitamins |
| Fructose | C02336 | C_6_H_12_O_6_ | Carbohydrates |
| Fucose | C01019 | C_6_H_12_O_5_ | Carbohydrates |
| Fumarate | C00122 | C_4_H_4_O_4_ | Carboxylic Acids |
| Glucose | C00031 | C_6_H_12_O_6_ | Carbohydrates |
| Glucuronate | C00191 | C_6_H_10_O_7_ | Carbohydrates |
| Glutamate | C00025 | C_5_H_9_NO_4_ | Amino acids |
| Glutamine | C00064 | C_5_H_10_N_2_O_3_ | Amino acids |
| Glycerol | C00116 | C_3_H_8_O_3_ | Carbohydrates |
| Glycine | C00037 | C_2_H_5_NO_2_ | Amino acids |
| Histidine | C00135 | C_6_H_9_N_3_O_2_ | Amino acids |
| Isoleucine | C00407 | C_6_H_13_NO_2_ | Amino acids |
| Leucine | C00123 | C_6_H_13_NO_2_ | Amino acids |
| Lysine | C00047 | C_6_H_14_N_2_O_2_ | Amino acids |
| Malate | C00149 | C_4_H_6_O_5_ | Organic acids |
| NAD+ | C00003 | C_21_H_28_N_7_O_14_P_2_ | Vitamins |
| O-Phosphocholine | C00588 | C_5_H_15_NO_4_P | Organic chemicals |
| Phenylalanine | C00079 | C_9_H_11_NO_2_ | Amino acids |
| Putrescine | C00134 | C_4_H_12_N_2_ | Organic chemicals |
| Serine | C00065 | C_3_H_7_NO_3_ | Amino acids |
| Serotonin | C00780 | C_10_H_12_N_2_O | Organic chemicals |
| sn-Glycero-3-phosphocholine | C00670 | C_8_H_21_NO_6_P | Carbohydrates |
| Succinate | C00042 | C_4_H_6_O_4_ | Organic acids |
| Sucrose | C00089 | C_12_H_22_O_11_ | Carbohydrates |
| Threonine | C00188 | C_4_H_9_NO_3_ | Amino acids |
| Tryptophan | C00078 | C_11_H_12_N_2_O_2_ | Amino acids |
| Tyrosine | C00082 | C_9_H_11_NO_3_ | Amino acids |
| UDP-glucose | C00029 | C_15_H_24_N_2_O_17_P_2_ | Vitamins |
| UMP | C00105 | C_9_H_13_N_2_O_9_P | Carbohydrates |
| Valine | C00183 | C_5_H_11_NO_2_ | Amino acids |
| γ-Aminobutyrate | C00334 | C_4_H_9_NO_2_ | Amino fatty acids |
