# Supplementary material for: Bacterial volatile organic compounds (VOCs) promote growth and induce metabolic changes in rice
Source: Front Plant Sci. 2023 Feb 9;13:1056082. doi: 10.3389/fpls.2022.1056082 (PMC9948655; doi:10.3389/fpls.2022.1056082)
Supplement: Supplementary file 15 [file Table_3.docx]

Supplementary Material

**Supplementary Table 3**. Fold change of the metabolites with significant differential abundance (t test FDR, p < 0.05) among treatments of rice co-cultivated with the bacterial isolates E.1b, IAT P4F9 and 1003-S-C1 and control plants.

| Compound | Treatment comparisons (Fold change) | | | | | |
| --- | --- | --- | --- | --- | --- | --- |
|  | E1b x CTL | E1b x 1003 | IAT x CTL | IAT x 1003 | 1003 x CTL | IAT x E1b |
| 2-Hydroxy-3-methylvalerate | * | 2.82 | * | -2.64 | 2.74 | * |
| 2-Hydroxyisocaproate | * | -10.59 | * | -10.59 | 10.59 | * |
| Alanine | * | * | 0.7 | * | * | * |
| AMP | * | * | * | * | * | * |
| Arginine | 1.73 | 1.2 | 1.48 | * | 0.53 | * |
| Ascorbate | * | * |  | * |  | * |
| Asparagine | 1.69 | * | 2.13 | * | 1.55 | * |
| Aspartate | * | * | 0.25 | * | * | * |
| Betaine | * | * | * | * | * | * |
| Caprate | * | * | 2.05 | 2.15 | * | 2.10 |
| Choline | * | * | * | * | * | * |
| Fructose | * | * | * | * | * | 1.55 |
| Fucose | -0.49 | -0.39 | * | * | * | * |
| Fumarate |  |  | 0.6 | * | 0.56 | * |
| Glucose | -1.39 | -1.52 | * | * | * | 1.79 |
| Glucuronate | * | * | * | * | * | * |
| Glutamate | * | -0.18 | * | -0.19 | 0.21 | * |
| Glutamine | 2.18 | * | 3.31 | 1.37 | 1.94 | 1.14 |
| Glycerol | * | * | 0.7 | 0.77 | * | 0.67 |
| Glycine | * | -0.5 | 1.21 | * | 0.77 | 0.93 |
| Histidine | 0.85 | * | 0.82 | * | 0.56 | * |
| Isoleucine | 1.29 | * | 1.41 | * | 0.91 | * |
| Leucine | 1.11 | * | 1.32 | 0.63 | 0.7 | * |
| Lysine | 1.38 | 0.87 | 1.55 | 1.04 | 0.51 | * |
| Malate | * | * | 1.52 | 0.84 | 0.67 | 1.24 |
| NAD+ | * | * | * | * | * | * |
| O-Phosphocholine | * | * | -0.17 | -0.3 | * | * |
| Phenylalanine | 1 | * | 1.08 | * | * | * |
| Putrescine | * | * | 1.02 | * | 0.72 | * |
| Serine | 0.7 | * | 0.73 | * | 0.7 | * |
| Serotonin | 0.75 | * | 1.29 | 0.93 | * | * |
| sn-Glycero-3-phosphocholine | * | * | * | * | * | * |
| Succinate | * | * | 1.19 | 1.19 | * | * |
| Sucrose | -0.55 | * | 0.77 | * | * | 1.32 |
| Threonine | 0.84 | * | 0.94 | * | 0.71 | * |
| Tryptophan | 1.44 | * | 1.65 | * | 0.93 | * |
| Tyrosine | 0.78 | * | 0.96 | * | 0.48 | * |
| UDP-glucose | * | * | -0.31 | -0.33 | * | * |
| UMP | * | * | * | * | * | * |
| Valine | 0.96 | * | 1.68 | 0.87 | 0.8 | 0.72 |
| γ-Aminobutyrate | 1.29 | * | 2.9 | 1.85 | 1.05 | * |

**Notes:** E1b = E.1b, IAT = IAT P4F9, 1003 = 1003-S-C1, CTL = Control; Red color = higher abundance, blue = lower abundance; * indicates no significant difference among the average of treatments of the respective comparison.
