# Supplementary material for: Bacterial volatile organic compounds (VOCs) promote growth and induce metabolic changes in rice
Source: Front Plant Sci. 2023 Feb 9;13:1056082. doi: 10.3389/fpls.2022.1056082 (PMC9948655; doi:10.3389/fpls.2022.1056082)
Supplement: Supplementary file 16 [file Table_4.docx]

Supplementary Material

**Supplementary Table 4.** PC loadings of the metabolites identified in shoot of rice co-cultivated with the bacterial isolates E.1b, IAT P4F9 and 1003-S-C1 and control plants.

| **Metabolite** | **PC loadings** | | |
| --- | --- | --- | --- |
|  | **PC1** | **PC2** | **PC3** |
| 2-Hydroxy-3-methylvalerate | -0,24623 | -0,02995 | 0,01085 |
| 2-Hydroxyisocaproate | -0,95655 | -0,04912 | -0,01925 |
| Alanine | 0,005924 | -0,07285 | 0,13968 |
| AMP | 0,013542 | -0,20974 | -0,08168 |
| Arginine | 0,046026 | -0,19601 | -0,27944 |
| Ascorbate | -0,00789 | -0,01703 | 0,093098 |
| Asparagine | -0,0249 | -0,28232 | -0,15795 |
| Aspartate | 0,003191 | -0,03021 | 0,027474 |
| Betaine | 0,025034 | 0,019678 | -0,02245 |
| Caprate | 0,079759 | -0,24165 | 0,30522 |
| Choline | 0,00423 | -0,06059 | -0,04327 |
| Fructose | -0,01679 | -0,08672 | 0,39331 |
| Fucose | -0,00782 | 0,016664 | 0,10906 |
| Fumarate | -0,01751 | -0,08125 | -0,05606 |
| Glucose | -0,0333 | -0,02991 | 0,48174 |
| Glucuronate | -0,00795 | -0,01168 | -0,01962 |
| Glutamate | -0,01782 | -0,00304 | -0,0165 |
| Glutamine | -0,0037 | -0,42875 | -0,04952 |
| Glycerol | 0,031305 | -0,07991 | 0,11623 |
| Glycine | -0,01994 | -0,1486 | 0,11235 |
| Histidine | -0,00052 | -0,11358 | -0,12579 |
| Isoleucine | 0,000199 | -0,19132 | -0,13008 |
| Leucine | 0,012256 | -0,17285 | -0,09273 |
| Lysine | 0,042517 | -0,20977 | -0,13306 |
| Malate | -0,00148 | -0,19107 | 0,12625 |
| NAD+ | 0,007221 | 0,026877 | 0,000359 |
| O-Phosphocholine | -0,01848 | 0,018773 | -0,01245 |
| Phenylalanine | 0,006203 | -0,15312 | -0,10152 |
| Putrescine | -0,04096 | -0,1223 | 0,2975 |
| Serine | -0,01973 | -0,09639 | -0,07339 |
| Serotonin | 0,031624 | -0,1628 | -0,0263 |
| sn-Glycero-3-phosphocholine | -0,02559 | 0,011676 | -0,12423 |
| Succinate | 0,049536 | -0,14956 | 0,041369 |
| Sucrose | 0,003435 | -0,08093 | 0,28933 |
| Threonine | -0,01 | -0,12557 | -0,09498 |
| Tryptophan | 0,010592 | -0,22247 | -0,14263 |
| Tyrosine | 0,010639 | -0,12859 | -0,05801 |
| UDP-glucose | -0,01829 | 0,042407 | -0,00144 |
| UMP | -0,00728 | -0,12931 | 0,045549 |
| Valine | 0,011013 | -0,21761 | 0,006385 |
| γ-Aminobutyrate | 0,037414 | -0,35495 | 0,1362 |
