# Supplementary material for: Bacterial volatile organic compounds (VOCs) promote growth and induce metabolic changes in rice
Source: Front Plant Sci. 2023 Feb 9;13:1056082. doi: 10.3389/fpls.2022.1056082 (PMC9948655; doi:10.3389/fpls.2022.1056082)
Supplement: Supplementary file 18 [file Table_6.docx]

Supplementary Material

**Supplementary Table 6.** Peak area of the volatile organic compounds (VOCs) identified in the volatilome of the bacterial isolates (E.1b, IAT P4F9 and 1003-S-C1) and *Escherichia coli* DH5α, when grown on LB medium.

| **Compound (IUPAC)** | **Chemical class** | **Formula** | **CAS** | **Bacteria** | | | |  |
| --- | --- | --- | --- | --- | --- | --- | --- | --- |
|  |  |  |  | **E.1b** | **IAT P4F9** | **1003-S-C1** | ***E. coli* DH5α** | |
| (5E)-6,10-dimethylundeca-5,9-dien-2-one | Terpenes | C_13_H_22_O | 3796-70-1 | 0.0003 | - | - | - | |
| (methyldisulfanyl)methane | Sulfides | C_2_H_6_S_2_ | 624-92-0 | 0.9458 | 0.0641 | - | - | |
| (methyltetrasulfanyl)methane | Sulfides | C_2_H_6_S_4_ | 5756-24-1 | 0.1092 | - | - | - | |
| (methyltrisulfanyl)methane | Sulfides | C_2_H_6_S_3_ | 3658-80-8 | 0.4876 | 0.0194 | - | 0.0432 | |
| 1-(1H-pyrrol-2-yl)ethanone | Pyrroles | C_6_H_7_NO | 1072-83-9 | 0.0028 | - | - | - | |
| 1-(2-aminophenyl)ethanone | Benzenoids | C_8_H_9_NO | 551-93-9 | 0.0096 | 0.0132 | - | - | |
| 1-(furan-2-yl)ethanone | Furan | C_6_H_6_O_2_ | 1192-62-7 | 0.0180 | - | - | - | |
| 1,2,3-trimethylbenzene | Benzenoids | C_9_H_12_ | 526-73-8 | 0.0026 | - | - | - | |
| 1,3-benzothiazole | Benzenoids | C_7_H_5_NS | 95-16-9 | 0.0017 | - | - | - | |
| 1-methoxy-4-methylbenzene | Benzenoids | C_8_H_10_O | 104-93-8 | - | 1.4077 | - | - | |
| 1-phenylpropan-2-one | Benzenoids | C_9_H_10_O | 103-79-7 | 0.0172 | - | - | - | |
| 2,4,6-trimethylpyridine | Pyridine | C_8_H_11_N | 108-75-8 | 0.1705 | - | - | - | |
| 2,4-dimethylfuran | Furan | C_6_H_8_O | 3710-43-8 | 0.0027 | - | - | - | |
| 2,5-dimethylpyrazine | Pyrazines | C_6_H_8_N_2_ | 123-32-0 | 0.0311 | - | - | - | |
| 2-ethyl-3,5,6-trimethylpyrazine | Pyrazines | C_9_H_14_N_2_ | 17398-16-2 | 0.0107 | - | - | - | |
| 2-ethyl-5-methylpyrazine | Pyrazines | C_7_H_10_N_2_ | 13360-64-0 | 0.0054 | - | - | - | |
| 2-ethylhexan-1-ol | Alcohols | C_8_H_18_O | 104-76-7 | 0.0054 | 0.0334 | 0.0100 | - | |
| 2-methoxyphenol | Benzenoids | C_7_H_8_O_2_ | 90-05-1 | - | 0.1664 | - | - | |
| 2-methylfuran | Furan | C_5_H_6_O | 534-22-5 | - | 0.0005 | - | - | |
| 2-phenylacetaldehyde | Benzenoids | C_8_H_8_O | 122-78-1 |  |  | 0.1499 |  | |
| 2-phenylethanol | Benzenoids | C_8_H_10_O | 60-12-8 | 0.0093 | 0.0151 | 0.7895 | 0.0713 | |
| 3-methylbutan-1-ol | Alcohols | C_5_H_12_O | 123-51-3 | 0.0018 | 0.1143 | 1.3321 | 0.0279 | |
| 3-methylbutyl acetate | Esters | C_7_H_14_O_2_ | 123-92-2 | - | - | 0.2253 | - | |
| 3-methylsulfanylpropan-1-ol | Sulfides | C_4_H_10_OS | 505-10-2 | - | 0.0106 | 0.2719 | - | |
| 4-methylquinazoline | Quinazoline | C_9_H_8_N_2_ | 700-46-9 | 0.0011 | - | - | - | |
| 6-methylheptan-2-one | Ketones | C_8_H_16_O | 928-68-7 | 0.0007 | - | - | - | |
| acetic acid | Acids | C_2_H_4_O_2_ | 64-19-7 | - | 0.0059 | - | - | |
| anisole | Benzenoids | C_7_H_8_O | 100-66-3 | - | 1.6407 | - | - | |
| benzonitrile | Nitrile | C_7_H_5_N | 100-47-0 | - | - | 0.0111 | - | |
| butan-2-one | Ketones | C_4_H_8_O | 78-93-3 | - | 0.0069 | 0.0018 | 0.0099 | |
| chloroform | Halogenated compounds | CHCl_3_ | 67-66-3 | 0.0006 | - | - | - | |
| decan-2-one | Ketones | C_10_H_20_O | 693-54-9 | 0.0035 | - | - | - | |
| dibutyl benzene-1,2-dicarboxylate | Esters | C_16_H_22_O_4_ | 84-74-2 | 0.0042 | - | - | - | |
| dodecan-2-one | Ketones | C_12_H_24_O | 6175-49-1 | 0.0047 | - | 0.0027 | - | |
| ethanol | Alcohols | C_2_H_6_O | 64-17-5 | - | 0.0020 | - | - | |
| ethylbenzene | Benzenoids | C_8_H_10_ | 100-41-4 | 0.0017 | - | - | - | |
| heptan-2-one | Ketones | C_7_H_14_O | 110-43-0 | 0.0088 | 0.0461 | - | - | |
| hexadecanoic acid | Acids | C_16_H_32_O_2_ | 57-10-3 | - | - | 0.0019 | - | |
| methanethiol | Thiols | CH_4_S | 74-93-1 | 0.0196 | 0.0279 | 0.0156 | 0.0351 | |
| methylsulfanylethane | Sulfides | C_3_H_8_S | 624-89-5 | 0.0001 | 0.0144 | 0.0033 |  | |
| methylsulfanylmethane | Sulfides | C_2_H_6_S | 75-18-3 | - | 0.0027 | 0.0008 | 0.0025 | |
| nonadecane | Alkanes | C_19_H_40_ | 629-92-5 | - | - | 0.0086 | - | |
| nonan-2-ol | Alcohols | C_9_H_20_O | 628-99-9 | 0.0271 | 0.0916 | 0.0085 | - | |
| nonan-2-one | Ketones | C_9_H_18_O | 821-55-6 | 0.1614 | 0.1847 | 0.0101 | 0.0319 | |
| octadecane | Alkanes | C_18_H_38_ | 593-45-3 | 0.0017 | - | - | - | |
| octan-2-one | Ketones | C_8_H_16_O | 111-13-7 | - | 0.0078 | - | - | |
| pentan-2-one | Ketones | C_5_H_10_O | 107-87-9 | - | 0.0030 | - | - | |
| phenol | Benzenoids | C_6_H_6_O | 108-95-2 | - | 0.5921 | - | - | |
| phenylmethanol | Benzenoids | C_7_H_8_O | 100-51-6 | - | 0.0587 | 0.2095 | 0.3104 | |
| S-methyl ethanethioate | Thioesters | C_3_H_6_OS | 1534-08-3 | 0.0036 | - | - | - | |
| toluene | Benzenoids | C_7_H_8_ | 108-88-3 | 0.0025 | 0.0064 | - | - | |
| tridecan-2-one | Ketones | C_13_H_26_O | 593-08-8 | 0.3907 | 0.0394 | 0.0344 | - | |
| undecan-2-ol | Alcohols | C_11_H_24_O | 1653-30-1 | 0.0242 | 0.0411 | 0.0168 | - | |
| undecan-2-one | Ketones | C_11_H_22_O | 112-12-9 | 0.0772 | 0.0285 | - | - | |
| undecanal | Aldehydes | C_11_H_22_O | 112-44-7 | 0.0182 | - | - | - | |
