# Supplementary material for: Bacterial volatile organic compounds (VOCs) promote growth and induce metabolic changes in rice
Source: Front Plant Sci. 2023 Feb 9;13:1056082. doi: 10.3389/fpls.2022.1056082 (PMC9948655; doi:10.3389/fpls.2022.1056082)
Supplement: Supplementary file 19 [file Table_7.docx]

Supplementary Material

**Supplementary Table 7.** PCA loadings of the volatile organic compounds (VOCs) identified in the volatilome of the bacterial isolates (E.1b, IAT P4F9 and 1003-S-C1) and *Escherichia coli* DH5α.

| **Compound** | **PC loadings** | | |
| --- | --- | --- | --- |
|  | **PC1** | **PC2** | **PC3** |
| (5E)-6,10-dimethylundeca-5,9-dien-2-one | 0.1136 | -0.0276 | -0.0125 |
| (methyldisulfanyl)methane | 0.2402 | 0.2055 | -0.0161 |
| (methyltetrasulfanyl)methane | 0.2263 | -0.0551 | -0.0248 |
| (methyltrisulfanyl)methane | 0.1510 | 0.0665 | 0.2890 |
| 1-(1H-pyrrol-2-yl)ethanone | 0.1568 | -0.0382 | -0.0172 |
| 1-(2-aminophenyl)ethanone | 0.1540 | 0.1881 | -0.0082 |
| 1-(furan-2-yl)ethanone | 0.1937 | -0.0472 | -0.0213 |
| 1,2,3-trimethylbenzene | 0.1556 | -0.0379 | -0.0171 |
| 1,3-benzothiazole | 0.1471 | -0.0358 | -0.0161 |
| 1-methoxy-4-methylbenzene | -0.0406 | 0.3475 | 0.0176 |
| 1-phenylpropan-2-one | 0.1925 | -0.0469 | -0.0211 |
| 2,4,6-trimethylpyridine | 0.2157 | -0.0529 | -0.0236 |
| 2,4-dimethylfuran | 0.1527 | -0.0373 | -0.0167 |
| 2,5-dimethylpyrazine | 0.2043 | -0.0498 | -0.0224 |
| 2-ethyl-3,5,6-trimethylpyrazine | 0.1835 | -0.0447 | -0.0201 |
| 2-ethyl-5-methylpyrazine | 0.1642 | -0.0402 | -0.0180 |
| 2-ethylhexan-1-ol | 0.0536 | 0.1280 | -0.2656 |
| 2-methoxyphenol | -0.0345 | 0.2952 | 0.0150 |
| 2-methylfuran | -0.0179 | 0.1530 | 0.0078 |
| 2-phenylacetaldehyde | -0.1119 | -0.1107 | -0.3358 |
| 2-phenylethanol | -0.0576 | -0.0494 | -0.0693 |
| 3-methylbutan-1-ol | -0.0945 | 0.0130 | -0.1048 |
| 3-methylbutyl acetate | -0.0807 | -0.0798 | -0.2420 |
| 3-methylsulfanylpropan-1-ol | -0.1439 | 0.1084 | -0.3416 |
| 4-methylquinazoline | 0.1378 | -0.0336 | -0.0151 |
| 6-methylheptan-2-one | 0.1301 | -0.0317 | -0.0143 |
| acetic acid | -0.0239 | 0.2042 | 0.0103 |
| anisole | -0.0410 | 0.3509 | 0.0178 |
| benzonitrile | -0.0873 | -0.0864 | -0.2618 |
| butan-2-one | -0.1648 | 0.0514 | 0.0670 |
| chloroform | 0.1261 | -0.0307 | -0.0138 |
| decan-2-one | 0.1614 | -0.0393 | -0.0177 |
| dibutyl benzene-1.2-dicarboxylate | 0.1630 | -0.0398 | -0.0179 |
| dodecan-2-one | 0.0944 | -0.1126 | -0.2364 |
| ethanol | -0.0218 | 0.1867 | 0.0095 |
| ethylbenzene | 0.1454 | -0.0355 | -0.0159 |
| heptan-2-one | 0.1490 | 0.2198 | -0.0064 |
| hexadecanoic acid | -0.0706 | -0.0698 | -0.2119 |
| methanethiol | -0.0042 | 0.0053 | 0.0242 |
| methylsulfanylethane | -0.0070 | 0.1360 | -0.2273 |
| methylsulfanylmethane | -0.1445 | 0.0502 | 0.0477 |
| nonadecane | -0.0798 | -0.0789 | -0.2396 |
| nonan-2-ol | 0.0838 | 0.1472 | -0.2631 |
| nonan-2-one | 0.0380 | 0.0463 | 0.0301 |
| octadecane | 0.1467 | -0.0357 | -0.0161 |
| octan-2-one | -0.0257 | 0.2200 | 0.0111 |
| pentan-2-one | -0.0230 | 0.1968 | 0.0100 |
| phenol | -0.0356 | 0.3044 | 0.0150 |
| phenylmethanol | -0.2409 | 0.0234 | 0.0344 |
| S-methyl ethanethioate | 0.1619 | -0.0394 | -0.0178 |
| toluene | 0.1294 | 0.1776 | -0.0061 |
| tridecan-2-one | 0.1258 | 0.1000 | -0.3094 |
| undecan-2-ol | 0.0776 | 0.1214 | -0.2831 |
| undecan-2-one | 0.1928 | 0.1971 | -0.0117 |
| undecanal | 0.1936 | -0.0472 | -0.0213 |
